# Effects of multiple stressors associated with agriculture on stream macroinvertebrate communities in a tropical catchment

**DOI:** 10.1371/journal.pone.0220528

**Published:** 2019-08-08

**Authors:** Aydeé Cornejo, Alan M. Tonin, Brenda Checa, Ana Raquel Tuñon, Diana Pérez, Enilda Coronado, Stefani González, Tomás Ríos, Pablo Macchi, Francisco Correa-Araneda, Luz Boyero

**Affiliations:** 1 Freshwater Macroinvertebrate Laboratory, Zoological Collection Dr. Eustorgio Mendez, Gorgas Memorial Institute for Health Studies (COZEM-ICGES), Panama City, Panama; 2 Doctoral Program in Natural Sciences with emphasis in Entomology, University of Panama, Panama City, Panama; 3 Department of Ecology, IB, Universidade de Brasília, Brasília, Distrito Federal, Brazil; 4 Plant Health Laboratory, Agricultural Development Ministry (MIDA), Panama City, Panama; 5 Environmental Quality Laboratory of the Ministry of Environment, Panama City, Panama; 6 Pacific Mariculture Station, Aquatic Resources Authority of Panama (ARAP), Panama City, Panama; 7 Museum of Freshwater Fish and Invertebrates, Autonomous University of Chiriquí, David, Panama; 8 Research Institute of Paleobiology and Geology, CONICET-National University of Río Negro, Río Negro, Argentina; 9 Center for Research in Environmental Toxicology and Agrobiotechnology of Comahue, CONICET-National University of Comahue, Buenos Aires, Argentina; 10 Unidad de Cambio Climático y Medio Ambiente, Instituto de Estudios de Hábitat (IEH), Facultad de Arquitectura y Construcción, Universidad Autónoma de Chile, Temuco, Chile; 11 Department of Plant Biology and Ecology, Faculty of Science and Technology, University of the Basque Country (UPV / EHU), Leioa, Spain; 12 IKERBASQUE, Bilbao, Spain; Universitat de Barcelona, SPAIN

## Abstract

Tropical forests are declining at unprecedented rates in favour of agriculture, and streams can be severely impacted due to effects of multiple stressors that have rarely been considered together in tropical studies. We studied the effects of multiple stressors associated with agricultural practices (pesticide toxicity, nutrient enrichment and habitat alteration–quantified as TU_max_, soluble reactive phosphorus concentration and sedimentation, respectively) on macroinvertebrate communities in a tropical catchment in Panama (13 stream sites sampled in 20 occasions from 2015 to 2017, with 260 samples in total). We examined how macroinvertebrate abundance, taxonomic richness, community composition and biotic indices (SPEAR and BMWP/PAN, which were specifically designed to detect pesticide toxicity and nutrient enrichment, respectively) varied depending on the studied stressors, considering their single and combined effects. Our analyses revealed significant effects of the studied stressors on macroinvertebrate communities, with two particular results that merit further attention: (1) the fact that pesticide toxicity affected BMWP/PAN, but not SPEAR, possibly because the former had been adapted for local fauna; and (2) that most stressors showed antagonistic interactions (i.e., lower combined effects than expected from their individual effects). These results highlight the need for toxicity bioassays with tropical species that allow adaptations of biotic indices, and of observational and manipulative studies exploring the combined effects of multiple stressors on tropical macroinvertebrate communities and ecosystems, in order to predict and manage future anthropogenic impacts on tropical streams.

## Introduction

Agriculture is one of the human activities with greatest impact on the Earth’s ecosystems [1]. Agricultural land now occupies c. 40% of the terrestrial surface [2], and it will most likely expand in the next few decades as a result of the higher demand of a larger global population [3]. This is particularly true for undeveloped countries, many of which are located in tropical regions [4]. Tropical forests are declining at unprecedented rates in favour of agriculture [5], and streams flowing through tropical agricultural catchments can be severely impacted [6].

Agriculture can alter stream communities and ecosystems for several reasons, including the increase in both inorganic and organic pollution as a result of the use of pesticides and fertilizers, respectively [7], and the alteration of riparian vegetation and physical habitat characteristics [8]. Streams are thus affected by multiple stressors [9,10], all related to agricultural practices, which should be considered together when assessing how agriculture impacts stream communities [11]. Such an approach, however, has rarely been used for the study of streams in the tropics, where information about effects of pesticides is scarce [6] and studies have generally considered the separate effects of nutrient enrichment [12] or altered habitat [13].

Effects of pesticides on tropical stream macroinvertebrates are largely unknown. Most information available on toxicity effects pertains to temperate species [14], which have been used to develop indices such as the widely used Species at Risk index (SPEARpesticides, hereafter SPEAR; [15]). Thus, while temperate studies have often found strong correlation between pesticides and SPEAR [16,17], the only tropical study using this approach, to our knowledge, did not find a similarly high correlation with SPEAR [6]. Effects of nutrient enrichment on stream macroinvertebrates have been generally assessed using indices such as the Biological Monitoring Working Party (BMWP), which is based on the sensitivity or tolerance of different macroinvertebrate families to nutrient enrichment [18]. The BMWP has been often used in the tropics, with adaptations of these indices accounting for differences in local fauna (e.g., [19]). Lastly, effects of altered habitat features (e.g., sedimentation, low dissolved oxygen, loss of riparian cover) on tropical stream macroinvertebrates have been assessed more often [20–22], but rarely within the context of agricultural practices.

We studied the effects of multiple stressors associated with agricultural practices on stream macroinvertebrate communities in a tropical catchment in Panama. We examined how macroinvertebrate abundance, taxonomic richness and biotic indices (SPEAR and BMWP) varied depending on pesticide toxicity (quantified as maximum toxic units, TU_max_), nutrient enrichment and habitat alteration, examining the single and combined effects of these stressors.

## Material and methods

### Study area and site selection

**Fig 1:**
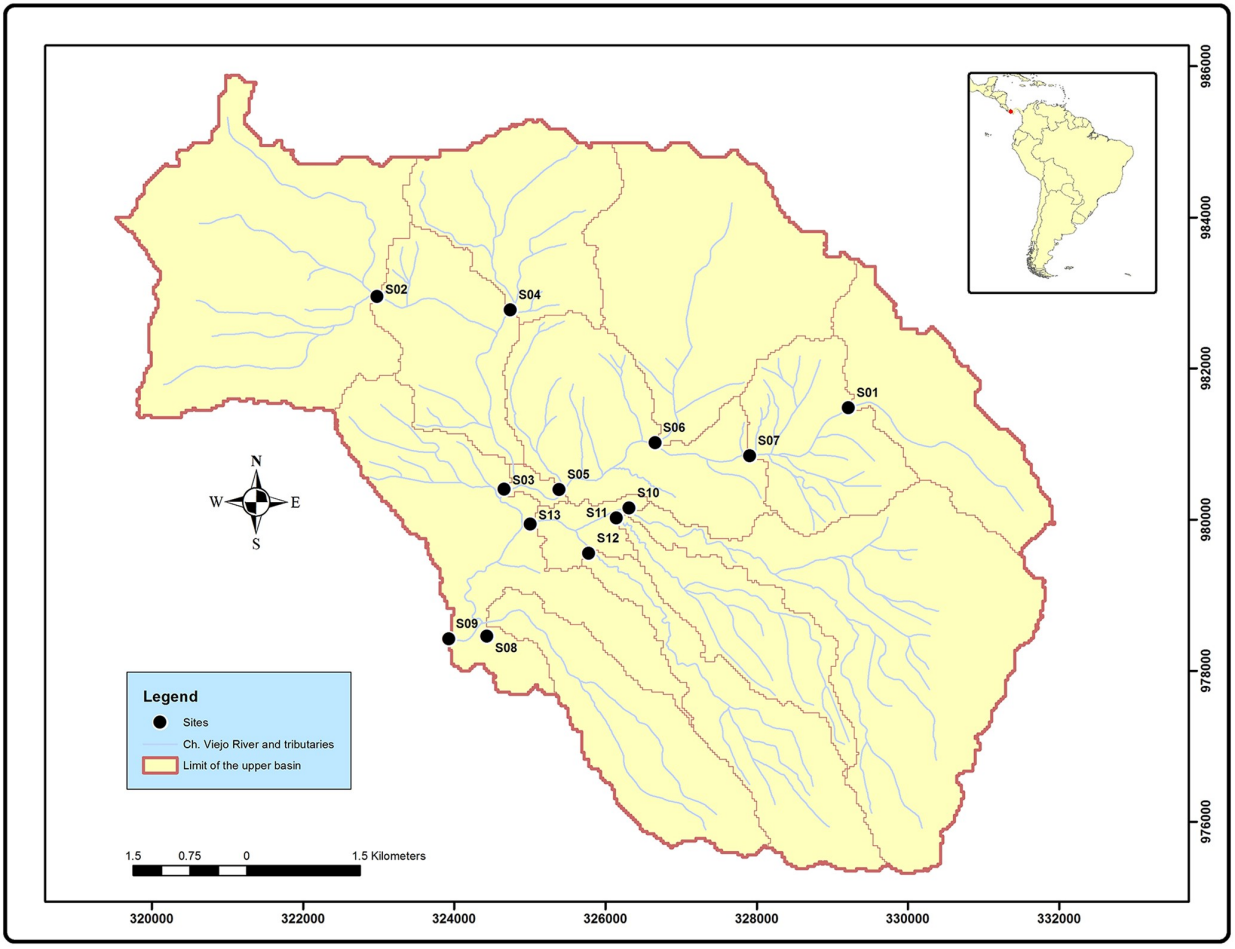
Location of study sites within the Chiriquí Viejo stream catchment in Panama.

Our study area was the upper catchment of the Chiriquí Viejo stream, located on the Pacific coast of western Panama (N 8°15'– 9°00', W 82°15'– 83°00'; Fig 1) [23]. Catchment area is 1,376 km^2^; total length of the main stream is 161 km; and maximum altitude is 3,474 m asl at Vocán Barú [24]. The climate is tropical with minimum, average and maximum annual temperatures of 17.8, 28.0 and 35.5 °C, respectively [25]. Total annual precipitation is 3,400 mm on average and up to 7,000 mm on the highlands, with 87.7% occurring in the rainy season (May-December) [24].

The study catchment is intensely used for agriculture, being one of the most productive areas in Panama [26]. The strong erosion, as a result of native vegetation removal, steep slopes and high precipitation, causes the progressive deterioration of the catchment, and stream water quality is affected by the entrance of fine sediment, pesticides and nutrients, the latter coming both from fertilizers and from the inefficient treatment of waste water in the area [27]. We conducted the study at 13 sites (Fig 1; S1 Table) from May 2015 to June 2017, with a total of 20 sampling campaigns at each site (March, May, August and October 2015, and monthly samplings from January to October 2016 and from January to June 2017; collecting permits issued be the Ministry of the Environment, Ref: SE/A-44-15, SC/A-5-16 and SE/A-42-17).

### Physico-chemical characterization

At each site we selected a 100-m long representative stream reach, where we (1) characterized the habitat; (2) measured several physico-chemical variables in situ, including substrate composition, coarse and fine particulate organic matter (CPOM > 1 mm; 0.5 μm < FPOM < 1 mm), and water chemistry; (3) collected water samples for further physico-chemical analyses and determination of pesticides; and (4) sampled macroinvertebrates.

We characterized the habitat using the rapid habitat assessment protocol developed by Barbour et al., [28] for the United States Environmental Protection Agency (EPA) for high-gradient streams. This consisted of qualifying 10 variables (epifaunal substrate/available cover, embeddedness, velocity/depth regime, sedimentation, channel flow status, channel alteration, frequency of riffles, bank stability, bank vegetative protection, and riparian vegetative zone width) using a numerical scale from 0 to 20 (maximum). Each variable was assessed independently, and their sum was assigned to one of four categories of habitat quality (i.e., optimal, suboptimal, marginal or poor).

Substrate composition was characterized visually as the proportion of different size classes of mineral substrate (boulder, cobble, gravel, coarse and fine sand, and clay) and CPOM and FPOM were quantified visually as the proportion of streambed covered by each type of organic matter [28]. We measured pH, temperature (°C), conductivity (μS cm^-1^), turbidity (mg L^-1^) and dissolved oxygen (% saturation) in situ using a multiparametric probe (YSI 556), and collected two sets of 2-Lwater samples from the mid column in middle of the stream, which were transported to the laboratory on ice. The first set of water samples was analysed at the Environmental Quality Laboratory of the Ministry of Environment (Panama) for concentrations (mg L^-1^) of total solids using a gravimetric method (SM 2540 B), and nitrate and soluble reactive phosphate (SRP) using spectrophotometric methods (SM 4500-NO_3_ B and SM 4500-P B5 and E) [29].

### Determination of pesticides

The second set of water samples was analysed for pesticides at the Plant Health Laboratory from the Agricultural Development Ministry (MIDA, Panama). A 2-L water sample was collected at each site from the middle of the stream and the mid column. Samples were immediately refrigerated and transported to the laboratory, and kept at 4 °C until analysis was performed within 24 h of receipt. Pesticides were determined using two methods: liquid-liquid microextraction [30] and direct injection [31]. The first method was used for organophosphates, organochlorines and pyrethroids; pesticides were extracted with ethyl acetate and residuals were quantified by gas chromatography and mass spectrophotometry (GC-MSMS; limit of quantification: 0.11 μg L^-1^). The second method was used for triazines, carbamates and other polar pesticides; samples were injected and analysed with high performance liquid chromatography and mass spectrophotometry (LC-MSMS; limit of quantification: 0.10 μg L^-1^) and electrospray ionization with dynamic acquisition (MRM mode), which avoids solid phase extraction. The percentage of recovery ranged between 70 and 110% (CV = 11%). Linearity was measured by the R^2^ coefficient for the individual pesticide calibration curves, always resulting in R^2^ ≥ 0.99. Each set of samples was analyzed in duplicate, simultaneously with a laboratory blank. To avoid matrix effects we used a matrix-matched calibration curve.

### Macroinvertebrate sampling and processing

Macroinvertebrates were kick sampled using a 30-cm wide D-net with a 0.5-mm mesh. At each site we took three 2-m long samples, which were subsequently pooled, with a total area of 1.8 m^2^ sampled per site. Samples were taken on a variety of habitats including mineral substrate, leaf litter patches and bank vegetation, in proportions similar to their presence in the stream. The net contents were transferred to a 0.5-mm mesh sieve and then to a white tray, where macroinvertebrates were preliminary sorted, and stones, leaves and wood discarded. The rest of the sample was introduced in labelled vials with 96% ethanol and transferred to the Freshwater Macroinvertebrate Laboratory at the COZEM-ICGES (Panama). Macroinvertebrates were sorted and identified to family level–which is the usual procedure to calculate the SPEAR and BMWP indices [18,32]–using identification keys for tropical taxa [33–36].

### Calculation of pesticide toxicity

In order to have a standard value of toxicity associated with pesticide concentrations measured at each site we used the Toxic Unit (TU) approach [37]. The TUs were given as maximum TU (TU_max_), a simple approach widely used in the literature [15;38,39]. To calculate TU_max_ we considered all pesticides found across samples at each site, excluding those below the quantification limit. Given that toxicity data for tropical stream macroinvertebrates are unavailable, we calculated TU_max_ based on data available for *Daphnia magna* [15] based on the following equation:

$${{TU}_{\left(D.magna\right)}={max}_{i=1}^{n}(\text{log}(C}_{i}/{LC50}_{i}))$$ (1)

where TU_(*D*. *magna*)_ is the TU_max_ of *n* pesticides detected in the study site, C_*i*_ is the concentration of pesticide *i* (μg L^-1^), and LC50_*i*_ is the 48 h acute median lethal concentration (μg L^-1^) reported for pesticide *i* in *D*. *magna*.

### Calculation of biotic indices

To calculate the SPEAR index, taxa were classified into species at risk (SPEAR) or species not at risk (SPEnotAR) according to several ecological and physiological traits [15], which were obtained from an online database (http://www.systemecology.eu/spear/spear-calculator/). The SPEAR value for each site was calculated as follows:

$$SPEAR=\frac{\sum_{i=1}^{n}\text{log}\left(x_{i}+1\right)\times y}{log(x_{i}+1)}\times 100$$ (2)

where *n* is the number of taxa, x_*i*_ is the abundance of taxon *i*, and *y* is 1 if taxon *i* is classified as SPEAR, otherwise 0.

The BMWP index is one of the most often used indices based on macroinvertebrates to assess nutrient enrichment in streams [40]. It was originally developed for the United Kingdom [18] and has been adapted to the local fauna of many countries, including Panama (BMWP/PAN; [27]). The BMWP/PAN was adapted based on tolerance to nutrient enrichment of local macroinvertebrate families, following the methods of Ruiz-Picos et al., [40]. The BMWP score at a given site is the sum of the individual scores of the families present at that site, which range from 1 (most tolerant families) to 9 (most sensitive families).

### Statistical analyses

All analyses were performed in R software, version 3.6.0 [41]. We first explored bivariate scatterplots and Pearson correlations to select the most relevant and uncorrelated environmental variables (r ≥ 0.70) to be used in further analyses (S1 Fig; [42]); these variables were TU_max_ (hereafter pesticide toxicity), SRP concentration (hereafter nutrient enrichment), the sediment deposition index (hereafter sedimentation index; inversely related to sedimentation), and water temperature (hereafter warming); other variables were discarded. Scatterplots and correlations were performed with the “chart.Correlation” function in PerformanceAnalytics package [43].

Secondly, we examined the individual and interactive effects of pesticide toxicity, nutrient enrichment and sedimentation index on macroinvertebrate abundance, taxonomic richness and biotic indices (SPEAR and BMWP/PAN), using linear mixed-effects models accounting for temporal autocorrelation. Warming influence was not considered in these models to avoid the complexity of a four-way interaction model, and because sedimentation was a better representation of habitat alteration (S2 Table). Models were first defined in terms of random structure, and a model selection procedure was used to identify interactions between predictors [42]. The optimal model random structure (i.e., the need for a variance structure, temporal correlation structure and/or random term) was defined by comparing models containing different terms using the Akaike Information Criterion corrected for sample size (AICc) (S3 Table). Final models were fit using the “lme” function (linear mixed effects), with site as a random term (except for the richness model, which lacked this component), temporal autocorrelation (ARMA correlation structure), and a variance structure (VarIdent in relation to site to control for different variances within sites).

Interactive effects were explored through five models, all containing the three predictors, but varying in the number of interactions. The null model (model 1) assumes no interactions between predictors (i.e., additive effects only); three models (models 2, 3 and 4) included pairwise interactions between nutrient enrichment and sedimentation index, pesticide toxicity and sedimentation index, or pesticide toxicity and nutrient enrichment; and one model (model 5) included all interactions, including the three-way interaction. The five models were compared using an AICc-based model selection approach, with the most plausible models being selected based on delta AICc (Δi; i.e., difference in AICc value relative to the best model) and Akaike weights (*w*i; i.e., the probability that a model is the best among the whole set of models). Residuals from each model were inspected to ensure there were no visual patterns and that linear model assumptions (i.e., independence and homogeneity assumptions) were not violated. Estimates and 95% confidence intervals for single predictors and their interactions were obtained using a model averaging approach, which averages the estimates of the retained models containing the parameter. Models were constructed, selected and averaged using nlme (“gls”, “lme”, “VarIdent” and “corARMA” functions; [44]) and MuMIn (“model.sel” and “model.avg” functions; [45]) packages.

Thirdly, we evaluated the effect of pesticide toxicity, nutrient enrichment and habitat alteration (sedimentation index and warming) on macroinvertebrate community composition using redundancy analysis (RDA; [46]), where the species dataset was predicted by the environmental dataset. Both datasets contained multiple samples taken over time and were averaged to produce a single value per site. Lastly, to quantify the amount of variability in macroinvertebrate community composition that can be attributed to each of the above environmental factors, as well as to their shared contribution (i.e., interactions between predictors), we used partial redundancy analysis [47]. The amount of variability explained by each factor and their shared contribution was based on adjusted R^2^ (R^2^_adj_), and their statistical significance tested through permutation tests (999 randomizations). Macroinvertebrate data were Hellinger-transformed prior to both procedures to provide an unbiased estimate of variance partitioning based on pRDA. Variance partitioning and permutation tests were performed using the “varpart” and “cca.anova” functions, respectively, both from the vegan package [48]. Results were presented using a Venn diagram, which was drawn on Inkscape, an open-source vector graphics editor.

## Results

### Physico-chemical characteristics

The study streams were circumneutral, pH being 7.5 on average (range across study sites: 6.7–7.9); water temperature was 16.1 °C (range: 13.3–18.2); conductivity was 44.2 μS s^-1^ (range: 8.3–111.4); turbidity was 21.6 mg L^-1^ (range: 2.3–82.7); dissolved oxygen saturation was 75.2% (72.9–77.3); total solids were 130.1 mg L^-1^ (range: 31.1–342.4); NO_3_ concentration was 12.6 mg L^-1^ (range: 1.5–33.5); and PO_4_ concentration was 0.23 mg L^-1^ (range: 0.04–0.53). The substrate was dominated by cobble at most sites, followed by gravel and coarse sand, and boulder was dominant at one site (S4 Table).

### Pesticides and TU_max_

We detected 29 pesticides in total, with 12 pesticides per site on average (range: 8–17). These included 19 insecticides (mostly chlorpyrifos and DDE-p.p', which were present at all study sites; and diazinon, HCB-gamma and mirex, present at 60% of sites), 9 fungicides (mostly carbendazim and iprobenfos present at 60% of sites) and one herbicide (metribuzin) (S5 Table). TU_max_ were -1.64 on average (range: 0.24– -4.46) (S6 Table).

### Macroinvertebrates

We collected 43,294 macoinvertebrate individuals from 57 families (S7 Table). The most common families were the Simuliidae (Diptera; 33.3% of total abundance), Baetidae (Ephemeroptera; 26.3%), Chironomidae (Diptera; 18.6%) and Physidae (Basommatophora; 4.6%). The average value of SPEAR was 28.2 (range: 0–73.4), and average BMWP/PAN was 28.5 (range: 1–103) (S8 Table).

### Interactive effects of pesticide toxicity, nutrient enrichment and habitat alteration on macroinvertebrate communities

**Fig 2:**
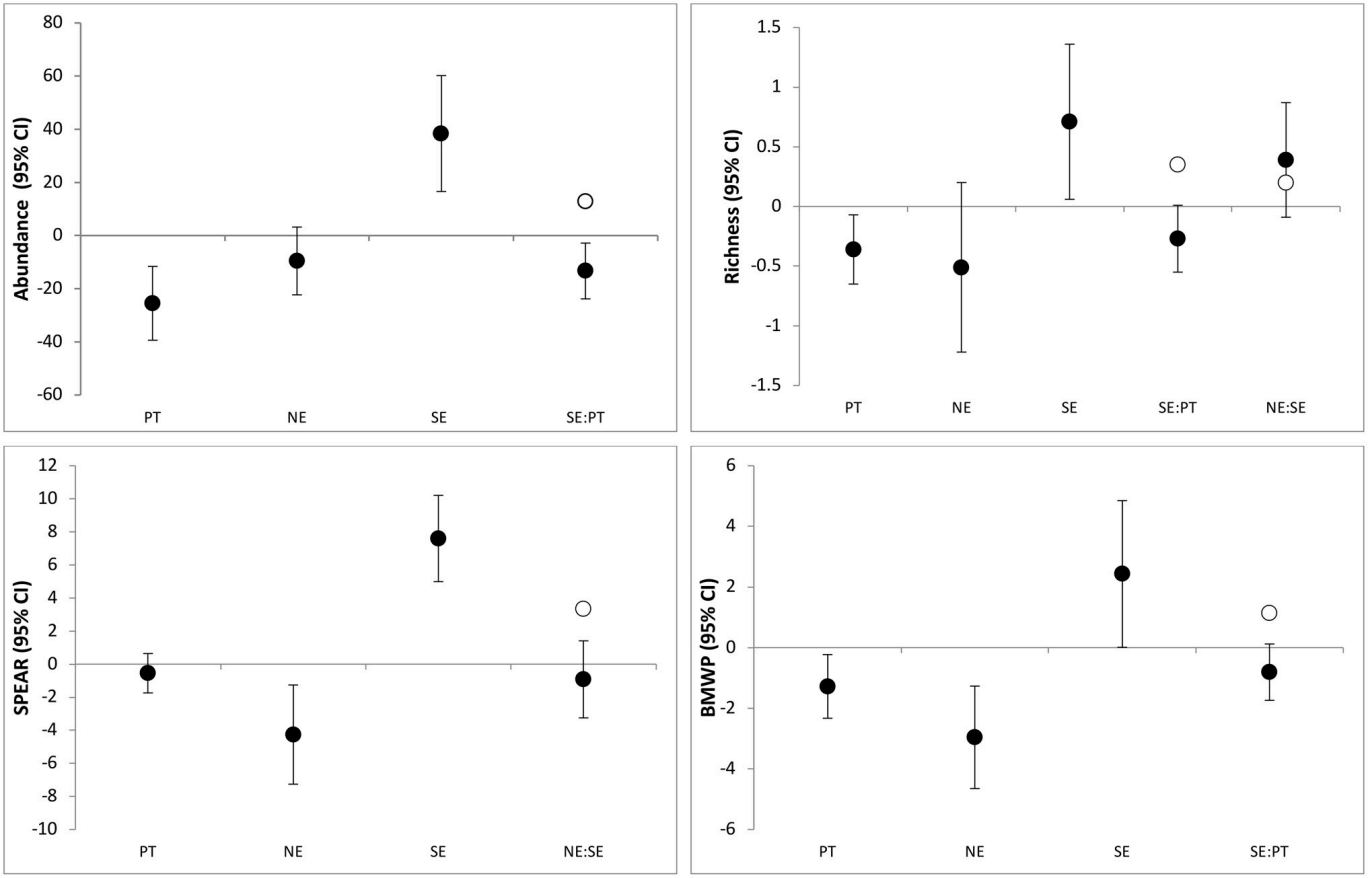
Estimates (slope of regression models) and 95% confidence intervals (CI, lower and upper whiskers) of individual stressors (pesticide toxicity, PT; nutrient enrichment, NE; and sedimentation index, SE, which was inverse to sedimentation) and their interactions present in the two most plausible models after model averaging (except for abundance, which was explained by a single model). Confidence intervals that intercept the zero line indicate no effect (i.e., do not reject the null hypothesis). Open circles denote the additive expectation for the interaction (i.e., the sum of the component individual effects); CIs containing the additive expectation indicate additive effects, while CIs not matching the additive expectation indicate either antagonistic effects (when the interaction does not surpass the effect of individual stressors) or synergistic effects (when it surpasses the effect of individual stressors).

The model selection procedure revealed that, in most cases, there were two best models explaining the observed patterns (~ 60% probability based on Akaike weights); the exception was abundance, which was explained by a single model with pairwise interactions. The SPEAR and BMWP indices were best explained by one additive model (i.e., without interactions) and one model containing pairwise interactions; the two most plausible models explaining richness contained pairwise interactions (Table 1; S9 Table). Overall, individual effects of pesticide toxicity and nutrient enrichment were negative, while the sedimentation index had a positive effect (indicating a negative effect of sedimentation, which was inverse to the index). The sedimentation index was the only factor individually affecting all the response variables; the individual effect of nutrient enrichment was important for both biotic indices, but not for abundance or richness; and pesticide toxicity individually affected all variables except SPEAR (Fig 2). The interaction between pesticide toxicity and sedimentation index was significant for abundance, richness and BMWP, always having a negative antagonistic effect (i.e., lower than predicted by the sum of individual effects); the interaction between nutrient enrichment and sedimentation index was important for richness and SPEAR, with a positive additive and a negative antagonistic effect, respectively (Fig 2).

**Table 1:** Summary of model selection testing for interactions between multiple stressors on macroinvertebrate abundance and richness and the SPEAR and BMWP indices, based on the Akaike information criterion corrected for sample size (AICc). Models are ordered from the best to the poorest fit according to Akaike weights (wi). K, number of estimated parameters for each model; Δi (delta AICc), difference in AICc value relative to the best model; wi, probability that a model is the best among the whole set of models. For each response variable, five models were constructed, which are ordered from the simplest model without interactions (model 1: null model, with no interactions) to the most complex one (model 5, containing the 3-way interaction). Models differ in the number of parameters according to the most parsimonious combination of structure and terms described in S9 Table. PT, pesticide toxicity (Tu_max_); SE, sedimentation index; NE, nutrient enrichment (SRP).

	Model	*K*	AICc	Δi	*wi*
	Abundance				
**(3)**	**PT + NE + SE + PT × SE**	**20**	**2771.8**	**0**	**0.657**
(1)	PT + NE + SE	19	2774.3	2.5	0.189
(2)	PT + NE + SE + NE **×** SE	20	2776.4	4.66	0.064
(4)	PT + NE + SE + PT **×** NE	20	2776.6	4.87	0.058
(5)	PT + NE + SE + PT **×** NE + PT **×** SE + NE × SE + PT **×** NE **×** SE	23	2777.8	6.01	0.033
	Richness				
**(3)**	**PT + NE + SE + PT × SE**	**8**	**1017.1**	**0**	**0.361**
**(2)**	**PT + NE + SE + NE × SE**	**8**	**1017.9**	**0.86**	**0.236**
(1)	PT + NE + SE	7	1018.4	1.28	0.191
(5)	PT + NE + SE + PT **×** NE + PT **×** SE + NE × SE + PT **×** NE **×** SE	11	1019	1.91	0.139
(4)	PT + NE + SE + PT **×** NE	8	1020.3	3.19	0.073
	SPEAR				
**(1)**	**PT + NE + SE**	**19**	**1730.8**	**0**	**0.489**
**(2)**	**PT + NE + SE + NE × SE**	**20**	**1732.7**	**1.85**	**0.194**
(4)	PT + NE + SE + PT **×** NE	20	1733	2.24	0.159
(3)	PT + NE + SE + PT × SE	20	1733.2	2.37	0.15
(5)	PT + NE + SE + PT **×** NE + PT **×** SE + NE × SE + PT **×** NE **×** SE	23	1738.8	7.96	0.009
	BMWP				
**(3)**	**PT + NE + SE + PT × SE**	**20**	**1638.1**	**0**	**0.378**
**(1)**	**PT + NE + SE**	**19**	**1638.4**	**0.36**	**0.316**
(2)	PT + NE + SE + NE × SE	20	1640.2	2.13	0.13
(4)	PT + NE + SE + PT **×** NE	20	1640.9	2.78	0.094
(5)	PT + NE + SE + PT **×** NE + PT **×** SE + NE × SE + PT **×** NE **×** SE	23	1641.1	3.05	0.082

**Fig 3:**
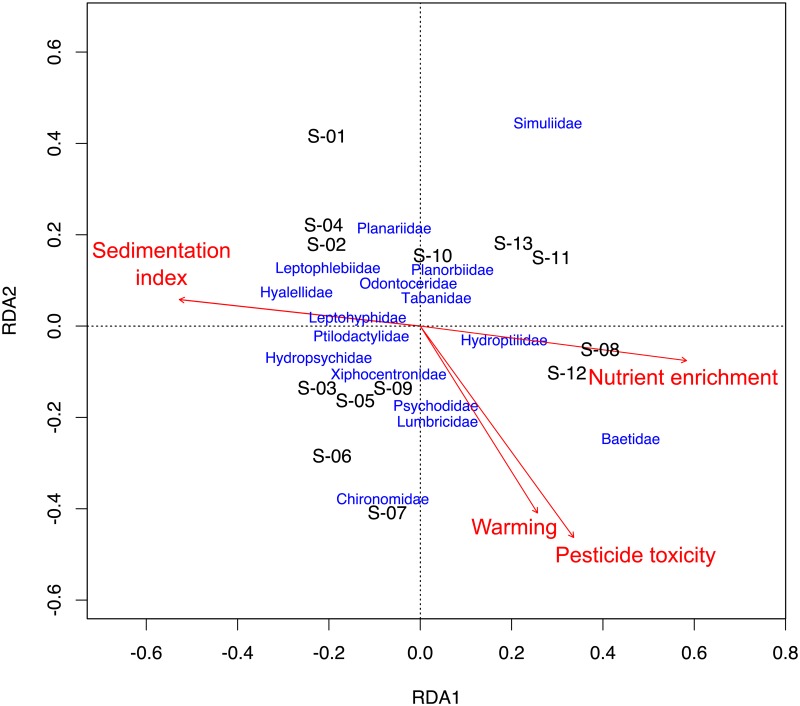
Redundancy analysis (RDA) exploring effects of pesticide toxicity (quantified as TU_max_), nutrient enrichment (SRP concentration) and habitat alteration (sedimentation index and warming) on macroinvertebrate community composition; RDA1 and RDA2 are the RDA axes, and S-01 to S-13 are the sampling sites.

All stressors explained 62% of variance in macroinvertebrate community composition. Nutrient enrichment and sedimentation were mostly related to RDA1 (both with positive relationships; the sedimentation index being inversely related to sedimentation), while pesticide toxicity and warming were related to RDA2 (both with negative relationships) (Fig 3). Thus, some taxa were related to sites with lower levels of pesticide toxicity, nutrient enrichment and habitat alteration (i.e., sites S-02, S-04 and S-10; Hyalellidae, Leptophyphidae, Leptophlebiidae, Planariidae, Planorbidae, Ptilodactylidae, Odontoceridae and Tabanidae) and others were associated to more impacted sites, that is, affected by nutrient enrichment and sedimentation (i.e., S-08 and S-12; Baetidae and Hydroptilidae) or higher levels of pesticide toxicity and warming (i.e., S-06 and S-07; Chironomidae, Lumbriculidae and Psychodidae).

**Fig 4:**
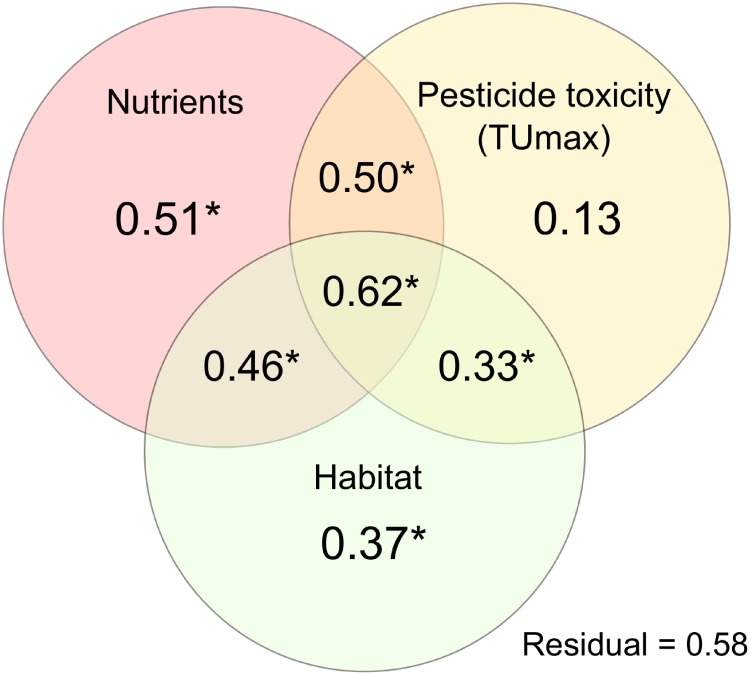
Partial redundancy analysis (pRDA). Quantifying the amount of variability in macroinvertebrate community composition attributable to pesticide toxicity (quantified as TU_max_), nutrient enrichment (SRP concentration) and habitat alteration (sediment deposition index–inversely related to sedimentation–and warming) and their shared contribution. The amount of variability explained by each factor or their shared contribution is based on R^2^_adj_; asterisks indicate significant results (at *p* < 0.05, based on 999 permutations).

The pRDA showed that a large proportion of variance in macroinvertebrate communities was driven by nutrient enrichment (R^2^_adj_ = 0.51) and habitat alteration (R^2^_adj_ = 0.37), while pesticide toxicity contributed to a lower proportion of variance (R^2^_adj_ = 0.13). The proportion of variance attributable to the combination of pesticide toxicity and nutrient enrichment (R^2^_adj_ = 0.50), nutrient enrichment and habitat alteration (R^2^_adj_ = 0.46) or the whole set of environmental predictors (R^2^_adj_ = 0.62), was lower than expected based on the sum of individual stressor effects (i.e., the additive expectation), indicating antagonistic effects. On the other hand, the combination of pesticide toxicity and habitat alteration (R^2^_adj_ = 0.33) was slightly higher than expected, suggesting a synergism between these two stressors (Table 2; Fig 4).

**Table 2:** Results of partial redundancy analysis (pRDA). Exploring the amount of variance in macroinvertebrate community composition explained by pesticide toxicity (TU_max_), nutrient enrichment (SRP) and habitat alteration (temperature and sedimentation index). We shown the degrees of freedom (df_model_, df_residual_), adjusted R^2^ (R^2^_adj_), associated *p*-values (*p*; after permutation tests using 999 randomizations), additive expectation (sum of R^2^_adj_ of individual stressors), and interaction type (A; antagonistic when R^2^_adj_ of interaction is lower than the sum of individual stressors; S, synergistic when R^2^_adj_ of interaction surpasses the sum of individual stressors).

Variables	df	R^2^_adj_	*p*	AD	Interaction
Pesticide toxicity (PT)	1, 11	0.13	0.114	-	-
Nutrient enrichment (NE)	1, 11	0.51	**0.006**	-	-
Habitat alteration (HA)	2, 10	0.37	**0.024**	-	-
PT × NE	2, 10	0.50	**0.011**	0.64	A
PT × HA	3, 9	0.55	**0.047**	0.50	S
NE × HA	3, 9	0.46	**0.021**	0.88	A
PT × NE × HA	4, 8	0.62	**0.049**	1.01	A
Residual	-	0.58	-	-	-

## Discussion

Assessing the effects of agricultural practices on tropical stream communities is an urgent challenge, given the fast conversion of tropical forests to agricultural land due to the rising demands of human populations [4,5,49]. Studies, however, are scarce and have only partially addressed this question, as they have focused on single stressors such as pesticide toxicity [6,50,51], nutrient enrichment [52] or habitat alteration, mainly deforestation [53–55] and sedimentation [20,56]. Our study is, to our knowledge, the first to assess the joint effect of multiple stressors associated with agriculture on tropical stream macroinvertebrate communities.

We demonstrated negative effects of the studied stressors (pesticide toxicity, nutrient enrichment, sedimentation and warming) on macroinvertebrate community descriptors and/or biotic indices. Sedimentation was the only factor with negative effects on all the variables; this factor has been shown to have large effects on tropical macroinvertebrates, which move downstream in response to increased sedimentation [20]. Abundance and richness were not affected by nutrient enrichment, in agreement with other tropical studies and possibly because other factors (e.g. light) limited primary productivity [12]. In contrast, abundance and richness were negatively affected by pesticide toxicity, an effect that has not been found elsewhere in the tropics [6,50]. The different stressors caused shifts in community composition, with some taxa being more tolerant to pesticide toxicity or warming (i.e., some dipterans and oligochaetes) and others to nutrient enrichment or sedimentation (i.e., some mayflies and caddisflies).

Interestingly, pesticide toxicity affected the BMWP/PAN but not the SPEAR index, which had been specifically designed to assess pesticide effects on macroinvertebrates [15]. This may be due to the fact that the SPEAR index is based on physiological traits associated with pesticide sensitivity in temperate species, which highlights the need for conducting biological toxicity tests with tropical macroinvertebrates, as these are likely to show different environmental sensitivities even at the taxonomic resolution of family [57]. This is supported by the fact that only studies in temperate areas have shown reduced levels of SPEAR with increased pesticide toxicity [15,58,59].

In our study, BMWP/PAN was affected by all the studied stressors, including pesticide toxicity. While the BMWP index was designed to assess effects of nutrient enrichment on macroinvertebrates [60], we used an index that had been adapted for local fauna (in contrast to SPEAR) and statistically calibrated [27,40], which may explain its significant response to all stressors. Temperate studies have also found an effect of pesticide toxicity on BMWP (but see [16,61]), while this has not been the case for other tropical studies using adapted versions of the index, such as the BMWP/COL [51].

Importantly, our analyses revealed interactive effects of different stressors that, in most cases, were antagonistic. Specifically, effects of pesticide toxicity or nutrient enrichment in combination with sedimentation on community descriptors and biotic indices were lower than expected from single effects, and the combined effects of most stressors on community composition were also antagonistic. These results suggest that assessing effects of stressors associated to agriculture individually can overestimate their overall effect, and highlights the importance of using a multi-stressor approach in real-context studies, because of the complex and often unpredictable interactions between stressors [10]. Our results are in accordance with a recent literature review, which found that additive and antagonistic interactions of multiple stressors were more prevalent than synergistic interactions [62]. Further studies should explore interactions of co-occurring stressors in the field, but also under controlled conditions where stressors can be easily manipulated (e.g., fully factorial designs in microcosms or mesocosms).

In summary, we provided novel evidence about negative effects of agricultural practices on tropical stream macroinvertebrate communities, which were affected by multiple stressors acting in combination. Our results highlight the need for (1) further tropical studies using a multi-stressor approach, including observational and manipulative studies assessing how macroinvertebrate communities and ecosystems respond to different combinations of stressors and; and (2) toxicity bioassays with tropical species that allow the adaptation of biotic indices to local fauna. Moreover, functional metrics such as leaf litter breakdown or other ecosystem processes can be useful tools for detecting ecosystem responses to nutrient enrichment [63,64], although these metrics also respond to other stressors and environmental drivers. Thus, the combined use of structural and functional metrics (e.g., biotic indices and ecosystem-level processes) could provide a more comprehensive assessment of the ecological effects of multiple stressors [65]. This is particularly needed in tropical areas, which are understudied and subject to rapid transformation by human activities [5], and whose responses compared to their temperate counterparts are difficult to predict [66].

## Supporting information

S1 Fig Correlogram showing pairwise Pearson correlations between variables sampled. Positive correlations are displayed in blue and negative correlations in red color. Color intensity and the size of the circle are proportional to the correlation coefficients. In the right side of the correlogram, the legend color shows the correlation coefficients and the corresponding colors. Only significant (p-value < 0.05) are displayed (blank spaces indicate non-significant correlations).
(DOCX)

S1 Table Coordinates of the sampling sites, sampling date and the number of samples collected at each site. (DOCX)

S2 Table Multiple pairwise Pearson correlations between sedimentation index, habitat quality and water temperature. (DOCX)

S3 Table Results of model selection to define the best random structure of models, in terms of variance structure, temporal correlation and random term. (DOCX)

S4 Table Habitat variables used for site characterization following Barbout et al. (1999); and physico-chemical variables (mean ± SE of 20 sampling campaigns). (DOCX)

S5 Table Mean (± SD) concentration (μg L-1) of pesticides in the water and total number of pesticides detected at each site in 20 sampling campaigns. (DOCX)

S6 Table TU_max_ values and concentrations of pesticides (μg/L) associated with campaign collects and sampling site. (DOCX)

S7 Table Mean (± SD) abundance of each macroinvertebrate taxon (mostly at family level) and of all macroinvertebrates, and total abundance and taxonomic richness, at each study site in 20 sampling campaigns. (DOCX)

S8 Table Average (±SD) score of biotic indices at each study site in 20 sampling campaigns. (DOCX)

S9 Table Results of the most plausible models (estimate and lower and upper 95% confidence intervals [CI]) after model selection. (DOCX)
