# Supplementary material for: Effects of multiple stressors associated with agriculture on stream macroinvertebrate communities in a tropical catchment
Source: PLoS One. 2019 Aug 8;14(8):e0220528. doi: 10.1371/journal.pone.0220528 (PMC6687280; doi:10.1371/journal.pone.0220528)
Supplement: S1 Fig — Positive correlations are displayed in blue and negative correlations in red color. Color intensity and the size of the circle are proportional to the correlation coefficients. In the right side of the correlogram, the legend color shows the correlation coefficients and the corresponding colors. Only significant (p-value < 0.05) are displayed (blank spaces indicate non-significant correlations). (DOCX) [file pone.0220528.s001.docx]

**Effects of multiple stressors associated with agriculture on stream macroinvertebrate communities in a tropical catchment**

Aydeé Cornejo, Alan M. Tonin, Brenda Checa, Ana Raquel Tuñon, Diana Pérez, Enilda Coronado, Stefani González, Tomás Ríos, Pablo Macchi, Francisco Correa-Araneda, Luz Boyero.

**Supporting information**

**S1 Fig 1**. Correlogram showing pairwise Pearson correlations between variables sampled. Positive correlations are displayed in blue and negative correlations in red color. Color intensity and the size of the circle are proportional to the correlation coefficients. In the right side of the correlogram, the legend color shows the correlation coefficients and the corresponding colors. Only significant (p-value < 0.05) are displayed (blank spaces indicate non-significant correlations).

**
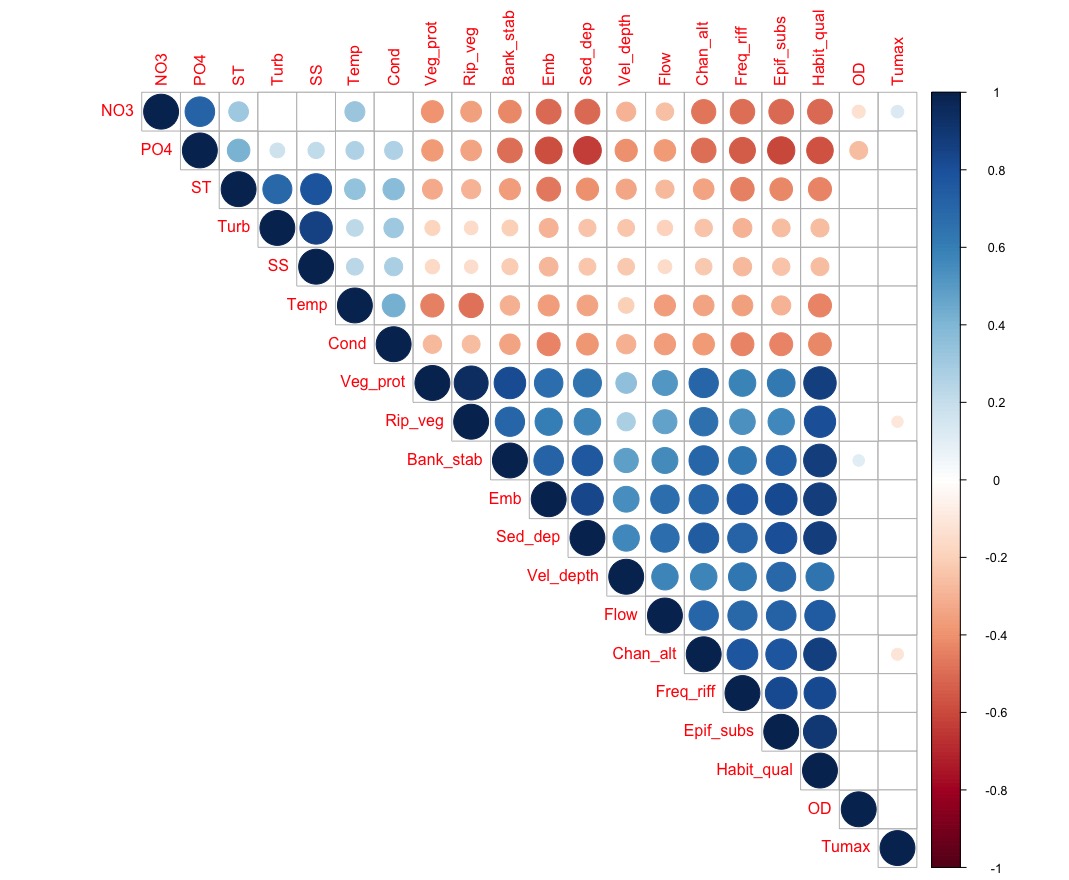
**
