# Supplementary material for: Effects of multiple stressors associated with agriculture on stream macroinvertebrate communities in a tropical catchment
Source: PLoS One. 2019 Aug 8;14(8):e0220528. doi: 10.1371/journal.pone.0220528 (PMC6687280; doi:10.1371/journal.pone.0220528)
Supplement: S1 Table — (DOCX) [file pone.0220528.s002.docx]

**Effects of multiple stressors associated with agriculture on stream macroinvertebrate communities in a tropical catchment**

Aydeé Cornejo, Alan M. Tonin, Brenda Checa, Ana Raquel Tuñon, Diana Pérez, Enilda Coronado, Stefani González, Tomás Ríos, Pablo Macchi, Francisco Correa-Araneda, Luz Boyero.

**Supporting information**

**S1 Table.** Coordinates of the sampling sites, sampling date and the number of samples collected at each site.

| Site Code | S-01 | S-02 | S-03 | S-04 | S-05 | S-06 | S-07 | S-08 | S-09 | S-10 | S-11 | S-12 | S-13 |
| --- | --- | --- | --- | --- | --- | --- | --- | --- | --- | --- | --- | --- | --- |
| Coordinates | 322984 | 324664 | 324744 | 325390 | 326658 | 327912 | 324436 | 323932 | 326313 | 326147 | 325782 | 325009 | 324811 |
|  | 982951 | 980401 | 982774 | 980398 | 981018 | 980842 | 978457 | 978421 | 980153 | 980023 | 979555 | 979941 | 980787 |
| Altitude (msnm) | 2323 | 2075 | 1820 | 2018 | 1843 | 1899 | 1510 | 1778 | 1708 | 1896 | 1887 | 1888 | 1846 |
| Phisic, chemical and microbiological sampling date (Number samples per year and total) | 2015-2016-2017 | 2015-2016-2017 | 2015-2016-2017 | 2015-2016-2017 | 2015-2016-2017 | 2015-2016-2017 | 2015-2016-2017 | 2015-2016-2017 | 2015-2016-2017 | 2015-2016-2017 | 2015-2016-2017 | 2015-2016-2017 | 2015-2016-2017 |
|  | (4-10-3) | (4-10-2) | (4-10-3) | (4-10-2) | (4-10-3) | (4-10-3) | (4-10-3) | (4-9-3) | (4-9-3) | (4-10-3) | (3-10-3) | (4-10-3) | (4-10-2) |
|  | 17 | 16 | 17 | 16 | 17 | 17 | 17 | 16 | 16 | 17 | 16 | 17 | 16 |
| Pesticides and macroinvertebrates sampling date (Number samples per year and total) | 2015-2016-2017 | 2015-2016-2017 | 2015-2016-2017 | 2015-2016-2017 | 2015-2016-2017 | 2015-2016-2017 | 2015-2016-2017 | 2015-2016-2017 | 2015-2016-2017 | 2015-2016-2017 | 2015-2016-2017 | 2015-2016-2017 | 2015-2016-2017 |
|  | (4-10-6) | (4-10-6) | (4-10-6) | (4-10-6) | (4-10-6) | (4-10-6) | (4-10-6) | (4-10-6) | (4-10-6) | (4-10-6) | (4-10-6) | (4-10-6) | (4-10-5) |
|  | 20 | 20 | 20 | 20 | 20 | 20 | 20 | 20 | 20 | 20 | 20 | 20 | 19 |
