# Supplementary material for: Effects of multiple stressors associated with agriculture on stream macroinvertebrate communities in a tropical catchment
Source: PLoS One. 2019 Aug 8;14(8):e0220528. doi: 10.1371/journal.pone.0220528 (PMC6687280; doi:10.1371/journal.pone.0220528)
Supplement: S2 Table — (DOCX) [file pone.0220528.s003.docx]

**Effects of multiple stressors associated with agriculture on stream macroinvertebrate communities in a tropical catchment**

Aydeé Cornejo, Alan M. Tonin, Brenda Checa, Ana Raquel Tuñon, Diana Pérez, Enilda Coronado, Stefani González, Tomás Ríos, Pablo Macchi, Francisco Correa-Araneda, Luz Boyero

**Supporting information**

**S2 Table.** Multiple pairwise Pearson correlations between sedimentation index, habitat quality and water temperature. Asterisks indicate statistical significant correlations (*P* < 0.001).

|  | **Sedimentation index** | **Temperature** | **Habitat quality** |
| --- | --- | --- | --- |
| **Sediment index** | - | -0.35 * | 0.87 * |
| **Temperature** | - | - | -0.44 * |
| **Habitat quality** | - | - | - |
