# Supplementary material for: Effects of multiple stressors associated with agriculture on stream macroinvertebrate communities in a tropical catchment
Source: PLoS One. 2019 Aug 8;14(8):e0220528. doi: 10.1371/journal.pone.0220528 (PMC6687280; doi:10.1371/journal.pone.0220528)
Supplement: S3 Table — (DOCX) [file pone.0220528.s004.docx]

**Effects of multiple stressors associated with agriculture on stream macroinvertebrate communities in a tropical catchment**

Aydeé Cornejo, Alan M. Tonin, Brenda Checa, Ana Raquel Tuñon, Diana Pérez, Enilda Coronado, Stefani González, Tomás Ríos, Pablo Macchi, Francisco Correa-Araneda, Luz Boyero.

**Supporting information**

**S3 Table**. Results of model selection to define the best random structure of models, in terms of variance structure, temporal correlation and random term. Candidate models were fitted using GLS (generalized least squares) and LME (linear mixed-effects) functions, one variance structure (VarIdent) in relation to site (to control for different variances within sites), a temporal correlation structure [ARMA(1,0)] and one random term (i.e., site). Models fitted with LME were adjusted for the number of iterations (Control iterations); maximum number of iterations for the optimization step set to 1,000 and number of iterations for the expectation–maximization algorithm set to 5,000. Models are ordered from the best to the poorest fit according to Akaike weights (wi). K, number of estimated parameters for each model; Δi (delta AICc), difference in AICc value relative to the best model; *w*i, probability that a model is the “best” among the whole set of models. Model complexity increases from the null to 5th model.

| Model | Model class | Variance structure | Temporal correlation | Control iterations | Random term | K | AICc | Δ_i_ | *w*_i_ |
| --- | --- | --- | --- | --- | --- | --- | --- | --- | --- |
| Abundance |  |  |  |  |  |  |  |  |  |
| (5) | LME | ✓ | ✓ | ✓ | ✓ | 23 | 2730.9 | 0 | 0.993 |
| (2) | GLS | ✓ | ✓ |  |  | 22 | 2740.8 | 9.94 | 0.007 |
| (1) | GLS | ✓ |  |  |  | 21 | 2754.0 | 23.05 | 0 |
| (3) | LME |  |  | ✓ | ✓ | 10 | 2799.1 | 68.16 | 0 |
| (4) | LME |  | ✓ | ✓ | ✓ | 11 | 2799.8 | 68.92 | 0 |
| (Null) | GLS |  |  |  |  | 9 | 2804.0 | 73.08 | 0 |
| Richness |  |  |  |  |  |  |  |  |  |
| (4) | LME |  | ✓ | ✓ | ✓ | 11 | 1027.2 | 0 | 0.968 |
| (3) | LME |  |  | ✓ | ✓ | 10 | 1034.6 | 7.33 | 0.025 |
| (5) | LME | ✓ | ✓ | ✓ | ✓ | 23 | 1037.0 | 9.8 | 0.007 |
| (1) | GLS | ✓ |  |  |  | 21 | 1057.2 | 29.99 | 0 |
| (2) | GLS | ✓ | ✓ |  |  | 22 | 1058.0 | 30.76 | 0 |
| (Null) | GLS |  |  |  |  | 9 | 1129.3 | 102.1 | 0 |
| SPEAR |  |  |  |  |  |  |  |  |  |
| (5) | LME | ✓ | ✓ | ✓ | ✓ | 23 | 1724.4 | 0 | 1 |
| (2) | GLS | ✓ | ✓ |  |  | 22 | 1745.2 | 20.8 | 0 |
| (4) | LME |  | ✓ | ✓ | ✓ | 11 | 1745.5 | 21.11 | 0 |
| (1) | GLS | ✓ |  |  |  | 21 | 1746.0 | 21.63 | 0 |
| (3) | LME |  |  | ✓ | ✓ | 10 | 1748.3 | 23.96 | 0 |
| (Null) | GLS |  |  |  |  | 9 | 1758.8 | 34.46 | 0 |
| BMWP |  |  |  |  |  |  |  |  |  |
| (5) | LME | ✓ | ✓ | ✓ | ✓ | 23 | 1628.7 | 0 | 0.576 |
| (4) | LME |  | ✓ | ✓ | ✓ | 11 | 1629.4 | 0.78 | 0.39 |
| (3) | LME |  |  | ✓ | ✓ | 10 | 1634.4 | 5.69 | 0.034 |
| (1) | GLS | ✓ |  |  |  | 21 | 1647.0 | 18.38 | 0 |
| (2) | GLS | ✓ | ✓ |  |  | 22 | 1649.3 | 20.66 | 0 |
| (Null) | GLS |  |  |  |  | 9 | 1748.4 | 119.78 | 0 |
