# Supplementary material for: Effects of multiple stressors associated with agriculture on stream macroinvertebrate communities in a tropical catchment
Source: PLoS One. 2019 Aug 8;14(8):e0220528. doi: 10.1371/journal.pone.0220528 (PMC6687280; doi:10.1371/journal.pone.0220528)
Supplement: S4 Table — (DOCX) [file pone.0220528.s005.docx]

**Effects of multiple stressors associated with agriculture on stream macroinvertebrate communities in a tropical catchment**

Aydeé Cornejo, Alan M. Tonin, Brenda Checa, Ana Raquel Tuñon, Diana Pérez, Enilda Coronado, Stefani González, Tomás Ríos, Pablo Macchi, Francisco Correa-Araneda, Luz Boyero.

**Supporting information**

**S4 Table.** Habitat variables used for site characterization following Barbout et al. (1999); and physico-chemical variables (mean ± SE of 20 sampling campaigns).

| **Site Code** | **S-01** | **S-02** | **S-03** | **S-04** | **S-05** | **S-06** | **S-07** | **S-08** | **S-09** | **S-10** | **S-11** | **S-12** | **S-13** |
| --- | --- | --- | --- | --- | --- | --- | --- | --- | --- | --- | --- | --- | --- |
| **Habitat variables** |  |  |  |  |  |  |  |  |  |  |  |  |  |
| Epifaunal substrate | 18.7 ± 0.8 | 13.2 ± 3.9 | 14.3 ± 1.9 | 16.2 ± 1.8 | 15.6 ± 1.1 | 14.2 ± 3.0 | 12.7 ± 3.6 | 6.1 ± 1.3 | 13.8 ± 2.2 | 12.3 ± 1.3 | 2.5 ± 0.5 | 3.7 ± 0.5 | 13.9 ± 2.3 |
| Embeddedness | 18.9 ± 0.9 | 15.7 ± 2.8 | 15.6 ± 1.4 | 17.0 ± 1.3 | 10.8 ± 1.9 | 15.6 ± 1.6 | 14.2 ± 2.5 | 5.7 ± 1.3 | 13.2 ± 3.0 | 10.7 ± 3.8 | 5.5 ± 0.9 | 6.1 ± 0.9 | 14.4 ± 2.6 |
| Velocity/depth combinations | 12.5 ± 1.5 | 12.7 ± 3.5 | 11.7 ± 2.4 | 12.4 ± 2.4 | 14.6 ± 3.7 | 12.5 ± 3.4 | 11.8 ± 3.0 | 7.5 ± 1.0 | 13.1 ± 2.2 | 10.7 ± 1.3 | 7.5 ± 1.2 | 6.9 ± 1.1 | 12.6 ± 3.3 |
| Sediment deposition | 18.2 ± 1.0 | 12.6 ± 2.8 | 14.3 ± 1.9 | 14.6 ± 2.4 | 10.8 ± 1.8 | 14.5 ± 2.2 | 13.8 ± 2.2 | 6.4 ± 1.6 | 12.0 ± 3.2 | 10.7 ± 2.1 | 4.6 ± 1.6 | 5.1 ± 2.2 | 10.6 ± 2.2 |
| Channel flow status | 17.7 ± 1.6 | 14.4 ± 3.0 | 12.6 ± 2.8 | 15.8 ± 2.8 | 13.8 ± 2.5 | 15.3 ± 2.6 | 14.9 ± 2.2 | 11.2 ± 0.5 | 14.1 ± 2.7 | 16.2 ± 1.0 | 8.1 ± 1.0 | 9.4 ± 2.2 | 14.7 ± 2.3 |
| Channel alteration | 20.0 ± 0 | 13.5 ± 2.0 | 15.3 ± 1.4 | 16.5 ± 1.4 | 15.5 ± 0.9 | 16.0 ± 1.9 | 13.2 ± 1.4 | 9.5 ± 1.4 | 13.3 ± 1.7 | 16.5 ± 1.0 | 10.3 ± 1.8 | 10.3 ± 2.2 | 14.4 ± 1.9 |
| Frequency of riffles | 18.2 ± 1.3 | 16.7 ± 1.0 | 16.0 ± 0.4 | 16.7 ± 2.0 | 16.6 ± 1.2 | 15.7 ± 1.2 | 15.8 ± 0.8 | 11.4 ± 0.6 | 16.2 ± 1.3 | 16.4 ± 0.9 | 11.4 ± 0.5 | 11.3 ± 0.4 | 16.1 ± 0.9 |
| Bank stability | 18.4 ± 0.8 | 4.0 ± 2.4 | 16.0 ± 0.7 | 16.3 ± 1.5 | 9.5 ± 1.2 | 12.0 ± 3.7 | 11.8 ± 3.9 | 3.0 ± 1.0 | 10.2 ± 3.0 | 7.1 ± 0.9 | 3.1 ± 0.9 | 2.9 ± 1.2 | 13.0 ± 3.5 |
| Bank vegetative protection | 20.0 ± 0 | 1.1 ± 0.7 | 8.2 ± 1.2 | 15.4 ± 0.6 | 5.4 ± 0.9 | 7.4 ± 0.9 | 5.6 ± 0.8 | 1.2 ± 0.4 | 10.9 ± 1.5 | 3.8 ± 0.4 | 2.0 ± 0 | 2.0 ± 0.0 | 12.1 ± 1.4 |
| Riparian vegetative zone width | 20.0 ± 0 | 0 | 3.9 ± 0.8 | 15.7 ± 0.6 | 1.4 ± 0.5 | 2.8 ± 0.4 | 2.2 ± 0.4 | 0 | 6.1 ± 1.7 | 2.8 ± 0.4 | 0 | 1.0 ± 0.0 | 7.2 ± 0.7 |
| Habitat quality | 182.5 ± 4.9 | 104.2 ± 13.7 | 127.7 ± 7.5 | 156.4 ± 8.4 | 113.8 ± 8.2 | 125.9 ± 14.7 | 115.2 ± 14.2 | 61.7 ± 4.4 | 125.2 ± 8.6 | 106.9 ± 7.3 | 54.8 ± 2.7 | 58.5 ± 2.8 | 129.1 ± 15.9 |
| **Inorganic substrate (%)** |  |  |  |  |  |  |  |  |  |  |  |  |  |
| Boulder (>256 mm) | 52.0 ± 12.4 | 22.0 ± 7.9 | 19.0 ± 9.7 | 18.0 ± 8.3 | 15.0 ± 5.1 | 29.0 ± 10.2 | 23.5 ± 5.6 | 16.3 ± 3.2 | 33.3 ± 7.7 | 19.5 ± 6.1 | 3.8 ± 4.3 | 3.5 ± 4.2 | 21.3 ± 11.0 |
| Cobble (64-256 mm) | 17.5 ± 4.4 | 39.5 ± 15.0 | 31.3 ± 13.0 | 34.0 ± 10.0 | 27.0 ± 6.6 | 31.8 ± 6.3 | 39.3 ± 16.4 | 37.3 ± 15.9 | 27.8 ± 13.8 | 16.3 ± 5.8 | 3.8 ± 4.3 | 6.5 ± 8.2 | 17.4 ± 12.8 |
| Gravel (2-64 mm) | 17.3 ± 9.4 | 26.3 ± 15.4 | 28.3 ± 13.3 | 34.5 ± 11.0 | 34.5 ± 7.6 | 23.0 ± 7.2 | 22.5 ± 18.5 | 9.3 ± 5.2 | 26.5 ± 15.7 | 25.0 ± 7.6 | 31.8 ± 14.8 | 10.5 ± 3.3 | 18.2 ± 11.7 |
| Coarse sand (0.06-2mm) | 7.3 ± 3.8 | 7.3 ± 2.6 | 13.5 ± 13.0 | 6.8 ± 2.5 | 13.5 ± 9.0 | 8.0 ± 3.0 | 6.0 ± 3.5 | 15.3 ± 7.0 | 6.5 ± 2.7 | 20.8 ± 5.7 | 38.0 ± 7.0 | 56.0 ± 10.5 | 22.4 ± 13.6 |
| Fine sand (0.004-0.006 mm) | 5.5 ± 2.8 | 4.3 ± 2.9 | 5.4 ± 2.4 | 5.3 ± 1.1 | 6.3 ± 2.2 | 5.5 ± 2.2 | 5.0 ± 0 | 1.0 ± 4.3 | 4.8 ± 2.0 | 12.3 ± 4.7 | 10.5 ± 4.8 | 14.0 ± 5.4 | 14.7 ± 9.9 |
| Clay (<0.004 mm) | 0.8 ± 1.8 | 1.5. ± 2.4 | 2.7 ± 3.4 | 1.5 ± 2.4 | 4.0 ± 3.8 | 2.8 ± 3.8 | 3.8 ± 2.2 | 12.0 ± 5.9 | 1.3 ± 2.2 | 6.8 ± 2.5 | 11.3 ± 6.3 | 9.5 ± 3.5 | 6.1 ± 4.6 |
| **Organic matter (%)** |  |  |  |  |  |  |  |  |  |  |  |  |  |
| CPOM | 72.0 ± 5.2 | 36.5 ± 4.9 | 63.5 ± 4.9 | 67.5 ± 4.4 | 63.0 ± 4.7 | 55.5 ± 8.3 | 49.0 ± 10.2 | 28.0 ± 4.1 | 42.0 ± 6.2 | 37.5 ± 4.4 | 30.5 ± 5.1 | 33.0 ± 4.5 | 46.8 ± 16.7 |
| FPOM | 28.0 ± 5.2 | 63.5 ± 4.9 | 36.5 ± 4.9 | 32.5 ± 4.4 | 37.0 ± 4.7 | 44.5 ± 8.3 | 51.0 ± 10.2 | 72.0 ± 4.1 | 58.0 ± 6.2 | 62.5 ± 4.4 | 69.5 ± 5.1 | 67.0 ± 4.5 | 53.2 ± 16.7 |
| **Physico-chemistry** |  |  |  |  |  |  |  |  |  |  |  |  |  |
| pH | 6.7 ± 0.7 | 7.2 ± 0.5 | 7.6 ± 0.4 | 7.2 ± 0.4 | 7.4 ± 0.5 | 7.3 ± 0.3 | 7.2 ± 0.4 | 7.9 ± 0.3 | 7.9 ± 0.3 | 7.6 ± 0.3 | 7.6 ± 0.4 | 7.5 ± 0.4 | 7.7 ± 0.4 |
| Temperature (°C) | 13.3 ± 0.6 | 14.8 ± 0.9 | 17.1 ± 1.3 | 14.2 ± 0.7 | 16.6 ± 1.0 | 16.7 ± 1.2 | 16.0 ± 0.9 | 17.9 ± 1.1 | 17.8 ± 1.2 | 16.6 ±1.3 | 14.9 ± 0.7 | 18.2 ± 1.8 | 15.7 ± 0.8 |
| Conductivity (μS cm^-1^) | 8.3 ± 8.0 | 13.9 ± 15.0 | 27.8 ±26.1 | 10.9 ± 12.2 | 29.8 ± 28.3 | 25.8 ± 25.5 | 31.9 ± 35.5 | 88.7 ± 80.5 | 42.0 ± 40.4 | 59.8 ± 56.5 | 58.8 ± 51.8 | 111.4 ± 105.6 | 65.0 ± 62.2 |
| Turbidity (mg L^-1^) | 2.3 ± 2.5 | 6.3 ± 13.6 | 10.3 ± 11.6 | 7.3 ±17.5 | 11.5 ± 16.6 | 13.2 ± 19.5 | 12.6 ± 14.9 | 82.7 ± 151.4 | 15.2 ± 10.2 | 12.2 ± 11.1 | 15.2 ± 30.9 | 76.3 ± 144.1 | 15.3 ± 12.1 |
| Dissolved oxygen (% saturation) | 74.4 ± 15.8 | 73.7 ± 17.1 | 75.8 ±17.8 | 74.4 ± 17.8 | 75.7 ± 17.4 | 77.3 ± 16.8 | 75.4 ± 15.6 | 76.4 ± 18.6 | 76.6 ± 18.1 | 72.9 ± 20.9 | 76.0 ± 18.5 | 74.1 ± 18.3 | 74.3 ± 18.6 |
| Total solids (mg L^-1^) | 31.1 ± 14.8 | 61.1 ± 24.0 | 66.5 ± 22.5 | 39.4 ± 25.3 | 69.7 ± 24.6 | 132.2 ± 254.4 | 100.4 ± 51.1 | 342.4 ± 306.1 | 119.8 ± 36.6 | 155.1 ± 49.3 | 140.6 ± 13.2 | 295.2 ± 142.7 | 168.1 ± 59.6 |
| NO3 (mg L^-1^) | 1.5 ± 2.3 | 2.1 ± 1.0 | 9.0 ± 8.6 | 1.6 ± 0.7 | 8.0 ± 4.5 | 7.8 ± 3.7 | 16.7 ± 9.0 | 30.2 ±15.1 | 13.5 ± 8.7 | 15.9 ± 6.9 | 8.9 ± 4.0 | 33.5 ± 13.0 | 15.6 ± 5.7 |
| PO4 (mg L^-1^) | 0.04 ± 0.04 | 0.05 ± 0.03 | 0.12 ± 0.07 | 0.04 ± 0.04 | 0.13 ± 0.07 | 0.11 ± 0.05 | 0.18 ± 0.11 | 0.53 ± 0.30 | 0.22 ± 0.13 | 0.25 ± 0.16 | 0.38 ± 0.19 | 0.51 ± 0.27 | 0.36 ± 0.20 |

**References**

Barbour MT, Gerritsen J, Snyder BD, Stribling JB. Rapid Bioassessment Protocols for Use in Streams and Wadeable Rivers: Periphyton, Benthic Macroinvertebrates and Fish, Second Edition. EPA 841-B-99-002. Washington, D.C. USA: U.S. Environmental Protection Agency, Office of Water, 1999.
