# Supplementary material for: Effects of multiple stressors associated with agriculture on stream macroinvertebrate communities in a tropical catchment
Source: PLoS One. 2019 Aug 8;14(8):e0220528. doi: 10.1371/journal.pone.0220528 (PMC6687280; doi:10.1371/journal.pone.0220528)
Supplement: S5 Table — (DOCX) [file pone.0220528.s006.docx]

**Effects of multiple stressors associated with agriculture on stream macroinvertebrate communities in a tropical catchment**

Aydeé Cornejo, Alan M. Tonin, Brenda Checa, Ana Raquel Tuñon, Diana Pérez, Enilda Coronado, Stefani González, Tomás Ríos, Pablo Macchi, Francisco Correa-Araneda, Luz Boyero.

**Supporting information**

**S5 Table.** Mean (± SD) concentration (µg L^-1^) of pesticides in the water and total number of pesticides detected at each site in 20 sampling campaigns. CAS are the Chemical Abstract Service registration numbers for pesticides.

| Pesticide type | CAS | Pesticide name | S-01 | S-02 | S-03 | S-04 | S-05 | S-06 | S-07 | S-08 | S-09 | S-10 | S-11 | S-12 | S-13 |
| --- | --- | --- | --- | --- | --- | --- | --- | --- | --- | --- | --- | --- | --- | --- | --- |
| Insecticide | 82657043 | bifenthrin | n.d. | 0.008 ± 0.034 | n.d. | 0.025 ± 0.084 | n.d. | 0.008 ± 0.034 | 0.008 ± 0.034 | n.d. | 0.018 ± 0.078 | 0.008 ± 0.034 | n.d. | n.d. | 0.008 ± 0.034 |
| Insecticide | 1563662 | carbofuran | n.d. | n.d. | 0.008 ± 0.034 | n.d. | n.d. | 0.018 ± 0.055 | n.d. | 0.010 ± 0.031 | n.d. | n.d. | n.d. | 0.009 ± 0.040 | n.d. |
| Insecticide | 52315078 | cypermethrin | n.d. | n.d. | n.d. | n.d. | n.d. | 0.018 ± 0.078 | 0.023 ± 0.101 | n.d. | 0.023 ± 0.101 | 0.019 ± 0.085 | 0.019 ± 0.083 | 0.018 ± 0.080 | n.d. |
| Insecticide | 2921882 | chlorpyrifos | 0.010 ± 0.042 | 0.010 ± 0.045 | 0.683 ± 2.965 | 0.010 ± 0.042 | 0.785 ± 3.403 | 0.026 ± 0.065 | 0.056 ± 0.117 | 0.630 ± 2.345 | 0.055 ± 0.115 | 0.010 ± 0.045 | 0.577 ± 2.335 | 0.034 ± 0.085 | 0.567 ± 2.399 |
| Insecticide | 72559 | DDE-p.p' | 0.027 ± 0.085 | 0.018 ± 0.080 | 0.044 ± 0.072 | 0.025 ± 0.079 | 0.029 ± 0.095 | 0.013 ± 0.056 | 0.023 ± 0.077 | 0.015 ± 0.067 | 0.016 ± 0.069 | 0.016 ± 0.069 | 0.028 ± 0.087 | 0.016 ± 0.069 | 0.026 ± 0.079 |
| Insecticide | 333415 | diazinon | 0.045 ± 0.164 | n.d. | 0.052 ± 0.145 | 0.108 ± 0.368 | 0.056 ± 0.155 | 0.024 ± 0.075 | 0.059 ± 0.159 | 0.052 ± 0.153 | 0.041 ± 0.144 | 0.033 ± 0.148 | 0.031 ± 0.136 | 0.076 ± 0.238 | 0.041 ± 0.141 |
| Insecticide | 60515 | dimethoate | n.d. | n.d. | n.d. | n.d. | 0.005 ± 0.022 | n.d. | n.d. | n.d. | n.d. | n.d. | n.d. | n.d. | n.d. |
| Insecticide | 1031078 | endosulfan sulfato | n.d. | n.d. | n.d. | n.d. | n.d. | n.d. | 0.023 ± 0.072 | 0.009 ± 0.038 | n.d. | n.d. | n.d. | n.d. | 0.014 ± 0.060 |
| Insecticide | 13194484 | ethoprophos | n.d. | n.d. | n.d. | n.d. | 0.078 ± 0.276 | 0.010 ± 0.042 | n.d. | n.d. | n.d. | n.d. | n.d. | 0.214 ± 0.893 | n.d. |
| Insecticide | 120068373 | fipronil | n.d. | n.d. | n.d. | n.d. | n.d. | n.d. | n.d. | 0.026 ± 0.116 | n.d. | n.d. | n.d. | n.d. | n.d. |
| Insecticide | 58899 | HCB-gamma | 0.018 ± 0.078 | 0.035 ± 0.078 | 0.018 ± 0.078 | 0.018 ± 0.078 | 0.018 ± 0.078 | 0.018 ± 0.080 | 0.018 ± 0.078 | n.d. | n.d. | 0.018 ± 0.078 | 0.026 ± 0.116 | n.d. | n.d. |
| Insecticide | 138261413 | imidacloprid | n.d. | n.d. | n.d. | n.d. | 0.006 ± 0.025 | n.d. | n.d. | 0.015 ± 0.049 | n.d. | 0 | n.d. | 0.019 ± 0.062 | 0.011 ± 0.048 |
| Insecticide | 91465086 | lambda-cyhalothrin | 0.041 ± 0.129 | n.d. | n.d. | n.d. | n.d. | 0.042 ± 0.131 | 0.025 ± 0.110 | 0.016 ± 0.072 | 0.016 ± 0.072 | 0.025 ± 0.110 | n.d. | 0.016 ± 0.072 | n.d. |
| Insecticide | 121755 | malathion | n.d. | n.d. | n.d. | n.d. | n.d. | n.d. | n.d. | 0.010 ± 0.045 | 0.011 ± 0.049 | n.d. | n.d. | n.d. | n.d. |
| Insecticide | 2385855 | mirex | 0.009 ± 0.038 | 0.009 ± 0.038 | 0.009 ± 0.038 | 0.025 ± 0.081 | 0.009 ± 0.038 | 0.009 ± 0.038 | 0.009 ± 0.038 | n.d. | n.d. | 0.009 ± 0.038 | n.d. | 0.016 ± 0.069 | 0.017 ± 0.076 |
| Insecticide | 23135220 | oxamyl | n.d. | n.d. | n.d. | n.d. | n.d. | 0.052 ± 0.233 | 0.087 ± 0.389 | n.d. | n.d. | 0.061 ± 0.271 | n.d. | 0.063 ± 0.282 | n.d. |
| Insecticide | 29232937 | pirimiphos-methyl | n.d. | n.d. | n.d. | n.d. | n.d. | n.d. | 0.024 ± 0.105 | n.d. | n.d. | n.d. | n.d. | n.d. | n.d. |
| Insecticide | 41198087 | profenofos | n.d. | n.d. | n.d. | n.d. | n.d. | n.d. | n.d. | n.d. | 0.014 ± 0.063 | n.d. | n.d. | n.d. | n.d. |
| Insecticide | 24017478 | triazophos | n.d. | 0.021 ± 0.094 | n.d. | n.d. | n.d. | n.d. | n.d. | n.d. | n.d. | n.d. | n.d. | 0.021 ± 0.094 | n.d. |
| Fungicide | 131860338 | azoxystrobin | n.d. | n.d. | n.d. | n.d. | n.d. | n.d. | 0.010 ± 0.045 | n.d. | n.d. | n.d. | n.d. | n.d. | n.d. |
| Fungicide | 10605217 | carbendazim | 0.003 ± 0.011 | 0.005 ± 0.015 | 0.003 ± 0.011 | 0.003 ± 0.011 | n.d. | 0.046 ± 0.141 | 0.101 ± 0.249 | 0.026 ± 0.114 | 0.081 ± 0.199 | 0.020 ± 0.076 | 0.025 ± 0.090 | 0.115 ± 0.284 | 0.079 ± 0.262 |
| Fungicide | 118741 | hexachlorobenzene | n.d. | 0.007 ± 0.031 | 0.007 ± 0.031 | n.d. | 0.032 ± 0.115 | n.d. | 0.007 ± 0.031 | n.d. | 0.023 ± 0.101 | n.d. | 0.023 ± 0.101 | n.d. | n.d. |
| Fungicide | 35554440 | imazalil | n.d. | 0.006 ± 0.028 | n.d. | n.d. | 0.006 ± 0.027 | n.d. | n.d. | 0.006 ± 0.028 | n.d. | 0.007 ± 0.029 | 0.006 ± 0.028 | n.d. | n.d. |
| Fungicide | 26087478 | iprobenfos | 0.007 ± 0.031 | 0.007 ± 0.031 | 0.007 ± 0.031 | 1.708 ± 7.603 | 0.008 ± 0.034 | 0.007 ± 0.031 | 0.044 ± 0.123 | n.d. | n.d. | 0.007 ± 0.031 | 1.697 ± 7.589 | n.d. | 0.011 ± 0.046 |
| Fungicide | 57837191 | metalaxyl | n.d. | n.d. | n.d. | n.d. | n.d. | n.d. | n.d. | n.d. | n.d. | 0.016 ± 0.072 | n.d. | n.d. | n.d. |
| Fungicide | 13457186 | pyrazophos | 0.017 ± 0.074 | n.d. | n.d. | n.d. | n.d. | 0.015 ± 0.065 | 0.014 ± 0.063 | n.d. | 0.015 ± 0.065 | n.d. | n.d. | n.d. | n.d. |
| Fungicide | 60207901 | propiconazole | n.d. | n.d. | n.d. | n.d. | n.d. | n.d. | 0.010 ± 0.042 | 0.009 ± 0.038 | 0.009 ± 0.038 | n.d. | n.d. | 0.013 ± 0.056 | n.d. |
| Fungicide | 107534963 | tebuconazole | 0.006 ± 0.025 | 0.009 ± 0.040 | 0.006 ± 0.011 | n.d. | n.d. | n.d. | n.d. | 0.006 ± 0.027 | n.d. | 0.003 ± 0.011 | n.d. | 0.003 ± 0.011 | 0.024 ± 0.106 |
| Herbicide | 21087649 | metribuzin | n.d. | n.d. | 0.125 ± 0.547 | n.d. | 0.173 ± 0.774 | n.d. | n.d. | 0.014 ± 0.051 | n.d. | n.d. | n.d. | 0.239 ± 0.736 | n.d. |
| # Pesticides | |  | **10** | **11** | **11** | **8** | **12** | **14** | **17** | **14** | **12** | **14** | **9** | **15** | **10** |

n.d.= not detected
