# Supplementary material for: Effects of multiple stressors associated with agriculture on stream macroinvertebrate communities in a tropical catchment
Source: PLoS One. 2019 Aug 8;14(8):e0220528. doi: 10.1371/journal.pone.0220528 (PMC6687280; doi:10.1371/journal.pone.0220528)
Supplement: S6 Table — (DOCX) [file pone.0220528.s007.docx]

**Effects of multiple stressors associated with agriculture on stream macroinvertebrate communities in a tropical catchment**

Aydeé Cornejo, Alan M. Tonin, Brenda Checa, Ana Raquel Tuñon, Diana Pérez, Enilda Coronado, Stefani González, Tomás Ríos, Pablo Macchi, Francisco Correa-Araneda, Luz Boyero.

**Supporting information**

**S6 Table.** TU_max_ values and concentrations of pesticides (µg/L) associated with campaign collects and sampling site.

| **Site** | **Date** | **Pesticides** | **Concentration**  **µg/L** | *Daphnia magna*  **Acute 48 hour LC50/EC50  µg/L^a^** | **TUmax** |
| --- | --- | --- | --- | --- | --- |
| S-01 | May.2015 | diazinon | 0.72 | 2 | -0.44 |
| S-01 | Feb.2016 | pyrazophos | 0.63 | 0.36 | 0.24 |
| S-01 | Apr.2016 | lambda-cyhalothrin | 0.33 | 1.4 | -0.63 |
| S-01 | Mar.2017 | tebuconazole | 0.11 | 750 | -3.83 |
| S-02 | May.2015 | DDE-p.p' | 0.36 | 1.9 | -0.72 |
| S-02 | Jan.2016 | hexachlorobenzene | 0.14 | 500 | -3.55 |
| S-02 | Feb.2016 | HCB-gamma | 0.35 | 250 | -2.85 |
| S-02 | Apr.2016 | bifenthrin | 0.15 | 0.86 | -0.76 |
| S-02 | Jul.2016 | triazophos | 0.42 | 12.92 | -1.49 |
| S-02 | Sep.2016 | imazalil | 0.126 | 3540 | -4.45 |
| S-03 | May.2015 | diazinon | 0.6 | 2 | -0.52 |
| S-03 | Aug.2015 | chlorpyrifos | 13.27 | 27.43 | -0.32 |
| S-03 | Jan.2016 | hexachlorobenzene | 0.14 | 500 | -3.55 |
| S-03 | Feb.2016 | HCB-gamma | 0.35 | 250 | -2.85 |
| S-03 | Apr.2016 | diazinon | 0.18 | 2 | -1.05 |
| S-03 | Jul.2016 | carbofuran | 0.15 | 38.6 | -2.41 |
| S-03 | Sep.2016 | diazinon | 0.25 | 2 | -0.90 |
| S-03 | Mar.2017 | metribuzin | 2.45 | 4180 | -3.23 |
| S-03 | Apr.2017 | carbendazim | 0.05 | 28.2 | -2.75 |
| S-04 | Mar.2015 | diazinon | 1.57 | 2 | -0.11 |
| S-04 | May.2015 | diazinon | 0.59 | 2 | -0.53 |
| S-04 | Aug.2015 | iprobenfos | 34.01 | 4200 | -2.09 |
| S-04 | Feb.2016 | HCB-gamma | 0.35 | 250 | -2.85 |
| S-04 | Apr.2016 | bifenthrin | 0.15 | 0.86 | -0.76 |
| S-05 | May.2015 | diazinon | 0.63 | 2 | -0.50 |
| S-05 | Aug.2015 | chlorpyrifos | 15.24 | 27.43 | -0.26 |
| S-05 | Jan.2016 | hexachlorobenzene | 0.14 | 500 | -3.55 |
| S-05 | Feb.2016 | HCB-gamma | 0.35 | 250 | -2.85 |
| S-05 | Apr.2016 | DDE-p.p' | 0.19 | 1.9 | -1.00 |
| S-05 | Jun.2016 | chlorpyrifos | 0.25 | 27.43 | -2.04 |
| S-05 | Aug.2016 | ethoprophos | 0.36 | 42.8 | -2.08 |
| S-05 | Sep.2016 | diazinon | 0.3 | 2 | -0.82 |
| S-05 | Mar.2017 | metribuzin | 3.46 | 4180 | -3.08 |
| S-06 | Feb.2016 | pyrazophos | 0.59 | 0.36 | 0.21 |
| S-06 | Apr.2016 | lambda-cyhalothrin | 0.33 | 1.4 | -0.63 |
| S-06 | Jun.2016 | carbofuran | 0.18 | 38.6 | -2.33 |
| S-06 | Jul.2016 | cypermethrin | 0.35 | 0.65 | -0.27 |
| S-06 | Aug.2016 | chlorpyrifos | 0.15 | 27.43 | -2.26 |
| S-06 | Sep.2016 | diazinon | 0.29 | 2 | -0.84 |
| S-06 | Feb.2017 | carbendazim | 0.42 | 28.2 | -1.83 |
| S-06 | Mar.2017 | carbendazim | 0.49 | 28.2 | -1.76 |
| S-06 | May.2017 | chlorpyrifos | 0.15 | 27.43 | -2.26 |
| S-07 | May.2015 | diazinon | 0.64 | 2 | -0.49 |
| S-07 | Jan.2016 | hexachlorobenzene | 0.14 | 500 | -3.55 |
| S-07 | Feb.2016 | pyrazophos | 0.58 | 0.36 | 0.21 |
| S-07 | Apr.2016 | bifenthrin | 0.15 | 0.86 | -0.76 |
| S-07 | Jun.2016 | propiconazole | 0.19 | 3200 | -4.23 |
| S-07 | Jul.2016 | cypermethrin | 0.45 | 0.65 | -0.16 |
| S-07 | Aug.2016 | chlorpyrifos | 0.32 | 27.43 | -1.93 |
| S-07 | Sep.2016 | diazinon | 0.29 | 2 | -0.84 |
| S-07 | Feb.2017 | carbendazim | 0.67 | 28.2 | -1.62 |
| S-07 | Mar.2017 | carbendazim | 0.79 | 28.2 | -1.55 |
| S-07 | Apr.2017 | carbendazim | 0.55 | 28.2 | -1.71 |
| S-07 | May.2017 | chlorpyrifos | 0.32 | 27.43 | -1.93 |
| S-08 | May.2015 | diazinon | 0.64 | 2 | -0.49 |
| S-08 | Aug.2015 | chlorpyrifos | 10.52 | 27.43 | -0.42 |
| S-08 | Mar.2016 | chlorpyrifos | 1.23 | 27.43 | -1.35 |
| S-08 | Apr.2016 | lambda-cyhalothrin | 0.32 | 1.4 | -0.64 |
| S-08 | Jun.2016 | carbofuran | 0.1 | 38.6 | -2.59 |
| S-08 | Jul.2016 | carbofuran | 0.1 | 38.6 | -2.59 |
| S-08 | Aug.2016 | chlorpyrifos | 0.16 | 27.43 | -2.23 |
| S-08 | Sep.2016 | diazinon | 0.27 | 2 | -0.87 |
| S-08 | Oct.2016 | malathion | 0.2 | 0.9 | -0.65 |
| S-08 | Apr.2017 | carbendazim | 0.51 | 28.2 | -1.74 |
| S-09 | May.2015 | diazinon | 0.63 | 2 | -0.50 |
| S-09 | Mar.2016 | chlorpyrifos | 0.25 | 27.43 | -2.04 |
| S-09 | Apr.2016 | diazinon | 0.18 | 2 | -1.05 |
| S-09 | Sep.2016 | chlorpyrifos | 0.31 | 27.43 | -1.95 |
| S-09 | Oct.2016 | pyrazophos | 0.29 | 0.36 | -0.09 |
| S-09 | Jan.2017 | carbendazim | 0.64 | 28.2 | -1.64 |
| S-09 | Mar.2017 | carbendazim | 0.45 | 28.2 | -1.80 |
| S-09 | Apr.2017 | carbendazim | 0.52 | 28.2 | -1.73 |
| S-10 | May.2015 | diazinon | 0.66 | 2 | -0.48 |
| S-10 | Feb.2016 | lambda-cyhalothrin | 0.49 | 1.4 | -0.46 |
| S-10 | Apr.2016 | bifenthrin | 0.15 | 0.86 | -0.76 |
| S-10 | Jul.2016 | cypermethrin | 0.38 | 0.65 | -0.23 |
| S-10 | Sep.2016 | imazalil | 0.131 | 3540 | -4.43 |
| S-10 | Jan.2017 | carbendazim | 0.34 | 28.2 | -1.92 |
| S-11 | Mar.2015 | chlorpyrifos | 0.74 | 27.43 | -1.57 |
| S-11 | May.2015 | diazinon | 0.61 | 2 | -0.52 |
| S-11 | Aug.2015 | chlorpyrifos | 10.47 | 27.43 | -0.42 |
| S-11 | Feb.2016 | chlorpyrifos | 0.14 | 27.43 | -2.29 |
| S-11 | Apr.2016 | DDE-p.p' | 0.25 | 1.9 | -0.88 |
| S-11 | Jul.2016 | cypermethrin | 0.37 | 0.65 | -0.24 |
| S-11 | Sep.2016 | imazalil | 0.123 | 3540 | -4.46 |
| S-11 | Apr.2017 | carbendazim | 0.4 | 28.2 | -1.85 |
| S-12 | Mar.2015 | mirex | 0.31 | 1970 | -3.80 |
| S-12 | May.2015 | diazinon | 0.63 | 2 | -0.50 |
| S-12 | Apr.2016 | lambda-cyhalothrin | 0.32 | 1.4 | -0.64 |
| S-12 | Jun.2016 | propiconazole | 0.25 | 3200 | -4.11 |
| S-12 | Jul.2016 | cypermethrin | 0.36 | 0.65 | -0.26 |
| S-12 | Aug.2016 | chlorpyrifos | 0.17 | 27.43 | -2.21 |
| S-12 | Sep.2016 | chlorpyrifos | 0.3 | 27.43 | -1.96 |
| S-12 | Feb.2017 | carbendazim | 1.11 | 28.2 | -1.40 |
| S-12 | Mar.2017 | carbendazim | 0.52 | 28.2 | -1.73 |
| S-12 | Apr.2017 | carbendazim | 0.52 | 28.2 | -1.73 |
| S-12 | Jun.2017 | diazinon | 0.89 | 2 | -0.35 |
| S-13 | Mar.2015 | mirex | 0.33 | 1970 | -3.78 |
| S-13 | May.2015 | diazinon | 0.6 | 2 | -0.52 |
| S-13 | Ago.2015 | chlorpyrifos | 10.47 | 27.43 | -0.42 |
| S-13 | Oct.2015 | endosulfan sulfate | 0.26 | 920 | -3.55 |
| S-13 | Mar.2016 | chlorpyrifos | 0.11 | 27.43 | -2.40 |
| S-13 | Apr.2016 | bifenthrin | 0.15 | 0.86 | -0.76 |
| S-13 | Jul.2016 | imidacloprid | 0.21 | 6029 | -4.46 |
| S-13 | Jan.2017 | carbendazim | 0.41 | 28.2 | -1.84 |
| S-13 | Mar.2017 | carbendazim | 1.09 | 28.2 | -1.41 |
| S-13 | Jun.2017 | tebuconazole | 0.46 | 750 | -3.21 |

Information on the LC_50_ or EC_50_ for *Daphnia magna* were obtained from the Ecotox database, USA-EPA (<https://cfpub.epa.gov/ecotox/>), except for hexachlorobenzene, metalaxyl and pyrazophos, which were obtained from the Pesticides Properties Database (PPDB) from the University of Hertfordshire (<https://sitem.herts.ac.uk/aeru/ppdb/>); both consulted on 05-02-2018.
