# Supplementary material for: Effects of multiple stressors associated with agriculture on stream macroinvertebrate communities in a tropical catchment
Source: PLoS One. 2019 Aug 8;14(8):e0220528. doi: 10.1371/journal.pone.0220528 (PMC6687280; doi:10.1371/journal.pone.0220528)
Supplement: S7 Table — (DOCX) [file pone.0220528.s008.docx]

**Effects of multiple stressors associated with agriculture on stream macroinvertebrate communities in a tropical catchment**

Aydeé Cornejo, Alan M. Tonin, Brenda Checa, Ana Raquel Tuñon, Diana Pérez, Enilda Coronado, Stefani González, Tomás Ríos, Pablo Macchi, Francisco Correa-Araneda, Luz Boyero.

**Supporting information**

**S7 Table.** Mean (± SD) abundance of each macroinvertebrate taxon (mostly at family level) and of all macroinvertebrates, and total abundance and taxonomic richness, at each study site in 20 sampling campaigns.

| TAXA | S-01 | S-02 | S-03 | S-04 | S-05 | S-06 | S-07 | S-08 | S-09 | S-10 | S-11 | S-12 | S-13 |
| --- | --- | --- | --- | --- | --- | --- | --- | --- | --- | --- | --- | --- | --- |
| PHYLUM PLATYHELMINTHES |  |  |  |  |  |  |  |  |  |  |  |  |  |
| Planariidae | 3.8 ± 3.6 | 2.3 ± 3.6 | 0.7 ± 1.3 | 1.8 ± 2.4 | 0.6 ± 1.2 | 1.0 ± 1.7 | 2.7 ± 5.5 | 0.1 ± 0.2 | 0.3 ± 0.7 | 2.9 ± 7.1 | 6.1 ± 5.4 | 0.2 ± 0.4 | 1.8 ± 4.0 |
| PHYLUM ANNELIDA |  |  |  |  |  |  |  |  |  |  |  |  |  |
| Lumbricidae | 0.8 ± 3.4 | 2.5 ± 11.2 | 0 | 0.3 ± 1.1 | 0 | 0 | 0.1 ± 0.5 | 0.2 ± 0.9 | 0 | 0.9 ± 3.8 | 0.3 ± 1.1 | 0.4 ± 1.8 | 0.4 ± 1.8 |
| Oligochaeta | 0 | 0 | 0 | 0 | 0.2 ± 0.5 | 0.1 ± 0.2 | 0 | 0 | 0 | 0.1 ± 0.2 | 0 | 0 | 0 |
| Tubificidae | 1.5 ± 2.2 | 4.1 ± 9.4 | 8.8 ± 22.6 | 1.5 ± 3.1 | 2.8 ± 4.5 | 5.4 ± 6.5 | 3.3 ± 4.0 | 3.1 ± 6.0 | 3.0 ± 5.7 | 6.0 ± 8.0 | 7.4 ± 8.9 | 3.3 ± 4.3 | 9.3 ± 19.0 |
| PHYLUM ARTHROPODA |  |  |  |  |  |  |  |  |  |  |  |  |  |
| SubPhylum Chelicerata |  |  |  |  |  |  |  |  |  |  |  |  |  |
| Class Arachnida |  |  |  |  |  |  |  |  |  |  |  |  |  |
| Entomobryidae | 0 | 0 | 0 | 0 | 0.1 ± 0.2 | 0 | 0.1 ± 0.5 | 0 | 0.2 ± 0.5 | 0.5 ± 1.2 | 0 | 0.1 ± 0.2 | 0.1 ± 0.3 |
| Hydrachnidia | 0.2 ± 0.4 | 1.0 ± 2.6 | 1.1 ± 2.5 | 0.3 ± 0.9 | 0.6 ± 1.2 | 1.3 ± 3.0 | 0.3 ± 0.7 | 0.1 ± 0.2 | 1.3 ± 2.5 | 0.5 ± 1.1 | 0.3 ± 0.6 | 0 | 0.7 ± 1.9 |
| SubPhylum Crustacea |  |  |  |  |  |  |  |  |  |  |  |  |  |
| Class Malacostraca |  |  |  |  |  |  |  |  |  |  |  |  |  |
| Order Amphipoda |  |  |  |  |  |  |  |  |  |  |  |  |  |
| Hyalellidae | 28.8 ± 21.7 | 0.1 ± 0.2 | 0.5 ± 2.0 | 1.1 ± 2.0 | 1.3 ± 5.6 | 0 | 0.1 ± 0.2 | 0.1 ± 0.2 | 0 | 0.1 ± 0.3 | 0 | 0.1 ± 0.2 | 0 |
| Order Isopoda | 0 | 0 | 0 | 0 | 0 | 0 | 0 | 0 | 0 | 0.1 ± 0.2 | 0.2 ± 0.7 | 0.1 ± 0.2 | 0 |
| Order Decapoda |  |  |  |  |  |  |  |  |  |  |  |  |  |
| Pseudothelphusidae | 0.2 ± 0.7 | 0 | 0 | 0 | 0 | 0 | 0 | 0 | 0.1 ± 0.2 | 0 | 0 | 0 | 0 |
| SubPhylum Hexapoda |  |  |  |  |  |  |  |  |  |  |  |  |  |
| Class Insecta |  |  |  |  |  |  |  |  |  |  |  |  |  |
| Order Ephemeroptera |  |  |  |  |  |  |  |  |  |  |  |  |  |
| Baetidae | 34.5 ± 31.1 | 123.9 ± 150.5 | 86.5 ± 72.1 | 46.80±67.94 | 52.1 ± 68.0 | 49.3 ± 46.7 | 30.9 ± 51.5 | 20.7 ± 57.1 | 68.7 ± 84.1 | 28.4 ± 40.4 | 1.0 ± 1.6 | 3.3 ± 9.8 | 24.0 ± 40.0 |
| Heptageniidae | 0.4 ± 0.9 | 0 | 0 | 0 | 0 | 0 | 0 | 0 | 0 | 0 | 0 | 0 | 0 |
| Leptohyphidae | 6.7 ± 6.2 | 0.1 ± 0.2 | 0 | 0.1 ± 0.2 | 0 | 0 | 0 | 0 | 0 | 0 | 0 | 0 | 0.1 ± 0.2 |
| Leptophlebiidae | 16.0 ± 10.8 | 0.3 ± 1.1 | 0 | 0.1 ± 0.2 | 0 | 0 | 0 | 0 | 0 | 0 | 0 | 0 | 0 |
| Order Odonata |  |  |  |  |  |  |  |  |  |  |  |  |  |
| Calopterygidae | 0 | 0 | 0 | 0.1 ± 0.2 | 0 | 0 | 0 | 0 | 0 | 0 | 0 | 0 | 0 |
| Coenagrionidae | 0 | 0 | 0 | 0.1 ± 0.3 | 0 | 0 | 0 | 0 | 0 | 0.1 ± 0.2 | 0 | 0 | 0 |
| Cordulegastridae | 0.1 ± 0.2 | 0 | 0 | 0 | 0 | 0 | 0 | 0 | 0 | 0 | 0 | 0 | 0 |
| Order Plecoptera |  |  |  |  |  |  |  |  |  |  |  |  |  |
| Perlidae | 7.8 ± 6.7 | 0.1 ± 0.2 | 0 | 0.9 ± 1.4 | 0 | 0 | 0 | 0 | 0 | 0 | 0 | 0 | 0 |
| Order Blattodea |  |  |  |  |  |  |  |  |  |  |  |  |  |
| Blaberidae | 0.1 ± 0.2 | 0 | 0 | 0 | 0 | 0 | 0 | 0 | 0 | 0 | 0 | 0 | 0 |
| Order Hemiptera |  |  |  |  |  |  |  |  |  |  |  |  |  |
| Gerridae | 0.7 ± 1.5 | 0.2 ± 0.7 | 0 | 0.5 ± 1.2 | 0 | 0 | 0 | 0 | 0 | 0 | 0 | 0 | 0 |
| Veliidae | 0.1 ± 0.2 | 0 | 0 | 0 | 0 | 0 | 0 | 0 | 0 | 0 | 0.1 ± 0.2 | 0 | 0 |
| Order Coleoptera |  |  |  |  |  |  |  |  |  |  |  |  |  |
| Elmidae | 0.6 ± 1.0 | 0 | 0 | 0.2 ± 0.4 | 0.1 ± 0.2 | 0.3 ± 1.1 | 0 | 0 | 0.1 ± 0.2 | 0 | 0 | 0.1 ± 0.2 | 0 |
| Hydrophilidae | 0 | 0 | 0 | 0.1 ± 0.2 | 0 | 0 | 0 | 0 | 0 | 0 | 0 | 0 | 0.3 ± 1.4 |
| Lampyridae | 0 | 0 | 0 | 0.1 ± 0.2 | 0 | 0 | 0 | 0 | 0 | 0 | 0 | 0 | 0 |
| Psephenidae | 1.5 ± 1.4 | 0 | 0 | 0 | 0 | 0 | 0 | 0 | 0.2 ± 0.7 | 0 | 0 | 0 | 0 |
| Ptilodactylidae | 14.7 ± 10.2 | 0.1 ± 0.2 | 0 | 1.2 ± 1.4 | 0.1 ± 0.2 | 0.1 ± 0.2 | 0.2 ± 0.5 | 0 | 0 | 0 | 0.1 ± 0.2 | 0 | 0 |
| Staphylinidae | 0.1 ± 0.2 | 0 | 0 | 0.1 ± 0.3 | 0 | 0 | 0 | 0.1 ± 0.5 | 0 | 0.1 ± 0.2 | 0.2 ± 0.7 | 0 | 0 |
| Order Megaloptera |  |  |  |  |  |  |  |  |  |  |  |  |  |
| Corydalidae | 0 | 0 | 0.1 ± 0.2 | 0 | 0 | 0 | 0 | 0 | 0 | 0 | 0 | 0 | 0 |
| Order Trichoptera |  |  |  |  |  |  |  |  |  |  |  |  |  |
| Calamoceratidae | 0.3 ± 1.0 | 0 | 0 | 0 | 0 | 0 | 0 | 0 | 0 | 0 | 0 | 0 | 0 |
| Glossosomatidae | 6.4 ± 10.2 | 4.2 ± 9.0 | 13.0 ± 16.6 | 1.5 ± 2.2 | 10.9 ± 14.1 | 15.7 ± 29.1 | 3.0 ± 6.4 | 0.8 ± 1.45 | 6.4 ±1 6.9 | 3.8 ± 7.0 | 0.1 ± 0.2 | 3.2 ± 14.1 | 8.4 ± 24.2 |
| Helicopsychidae | 0.4 ± 0.6 | 0.1 ± 0.2 | 0 | 0 | 0 | 0 | 0 | 0 | 0 | 0 | 0 | 0 | 0 |
| Hydrobiosidae | 5.5 ± 6.4 | 0.2 ± 0.7 | 0 | 0 | 0 | 0 | 0 | 0 | 0 | 0 | 0 | 0 | 0 |
| Hydropsychidae | 16.7 ± 14.4 | 0.5 ± 2.0 | 0 | 2.3 ± 3.5 | 0 | 0 | 0 | 0 | 0 | 0 | 0 | 0 | 0 |
| Hydroptilidae | 0.4 ± 1.4 | 8.2 ± 17.0 | 1.1 ± 2.6 | 3.2 ± 9.2 | 2.0 ± 6.45 | 9.2 ± 28.9 | 0.7 ± 1.5 | 0.1 ± 0.2 | 0.4 ± 1.1 | 3.3 ± 6.8 | 0.9 ± 2.1 | 0.1 ± 0.5 | 1.1 ± 1.5 |
| Lepidostomatidae | 0.6 ± 0.9 | 0 | 0 | 0.10 ± 0.3 | 0.1 ± 0.2 | 0 | 0.1 ± 0.2 | 0 | 0 | 1.8 ± 6.9 | 0 | 0 | 0.2 ± 0.7 |
| Leptoceridae | 0.3 ± 0.8 | 0.1 ± 0.5 | 0 | 0 | 0 | 0 | 0.1 ± 0.2 | 0 | 0 | 0 | 0 | 0 | 0.1 ± 0.2 |
| Odontoceridae | 0.1 ± 0.2 | 0 | 0 | 0 | 0 | 0.1 ± 0.2 | 0 | 0 | 0 | 0 | 0 | 0 | 0 |
| Philopotamidae | 1.3 ± 2.3 | 0 | 0 | 0 | 0 | 0 | 0 | 0 | 0 | 0 | 0 | 0 | 0 |
| Polycentropodidae | 1.5 ± 2.2 | 0.2 ± 0.5 | 0 | 0.1 ± 0.2 | 0 | 0 | 0.1 ± 0.2 | 0 | 0 | 0 | 0 | 0 | 0 |
| Xiphocentronidae | 0.3 ± 1.3 | 0 | 0 | 0.3 ± 0.7 | 0 | 0 | 0 | 0 | 0 | 0 | 0 | 0 | 0 |
| Order Lepidoptera |  |  |  |  |  |  |  |  |  |  |  |  |  |
| Cosmopterygidae | 0 | 0.1 ± 0.2 | 0 | 0 | 0 | 0.1 ± 0.2 | 0 | 0 | 0.1 ± 0.2 | 0 | 0 | 0 | 0 |
| Order Diptera |  |  |  |  |  |  |  |  |  |  |  |  |  |
| Blepharoceridae | 0.1 ± 0.2 | 0.1 ± 0.2 | 0 | 0 | 0 | 0 | 0 | 0 | 0 | 0 | 0 | 0 | 0 |
| Ceratopogonidae | 0.6 ± 1.1 | 0 | 0 | 0.1 ± 0.2 | 0 | 0 | 0.3. ± 1.1 | 0.1 ± 0.2 | 0 | 0 | 0 | 0 | 0 |
| Chironomidae | 33.3 ± 34.6 | 39.2 ± 84.0 | 33.2 ± 50.2 | 28.0 ± 46.3 | 25.5 ± 26.9 | 42.2 ± 42.2 | 50.0 ± 65.0 | 17.6 ± 23.2 | 40.6 ± 74.2 | 26.1 ± 32.2 | 35.0 ± 65.5 | 13.8 ± 21.1 | 19.3 ± 32.1 |
| Dixidae | 0.1 ± 0.3 | 0 | 0 | 0 | 0 | 0 | 0 | 0 | 0 | 0 | 0 | 0 | 0 |
| Empididae | 0.8 ± 1.2 | 3.0 ± 5.2 | 2.8 ± 7.1 | 1.1 ± 2.2 | 1.4 ± 2.5 | 1.3 ± 2.9 | 1.0 ± 1.9 | 1.7 ± 6.1 | 2.0 ± 4.5 | 0.6 ± 1.5 | 0.1 ± 0.2 | 0 | 0.8 ± 2.1 |
| Muscidae | 0 | 0.1 ± 0.2 | 0.1 ± 0.3 | 0.1 ± 0.2 | 0 | 0 | 0.1 ± 0.2 | 0 | 0 | 0 | 0.2 ± 0.5 | 0 | 0 |
| Psychodidae | 0.2 ±0.7 | 1.0 ± 1.6 | 4.2 ± 4.8 | 0.4 ± 0.8 | 3.3 ± 6.0 | 3.0 ± 3.9 | 0.9 ± 1.4 | 0.4 ± 0.9 | 2.9 ± 4.2 | 0.5 ± 1.1 | 0 | 0.2 ± 0.6 | 1.2 ± 2.8 |
| Simuliidae | 13.3 ± 12.8 | 58.5 ± 85.5 | 157.7 ± 185.9 | 23.2 ± 52.4 | 119.3 ±170.2 | 78.7 ± 73.2 | 91.2 ± 107.6 | 3.2 ± 6.4 | 40.5 ± 55.0 | 87.6 ± 111.6 | 16.3 ± 32.9 | 0.3 ± 0.7 | 32.1 ± 57.1 |
| Stratiomyidae | 0 | 0 | 0.1 ± 0.5 | 0.1 ± 0.2 | 0.2 ± 0.5 | 0.2 ± 0.7 | 0.1 ± 0.5 | 0 | 0.1 ± 0.2 | 0 | 0 | 0.1 ± 0.2 | 0.1 ± 0.5 |
| Tabanidae | 0 | 0.1 ± 0.5 | 0 | 0.1 ± 0.5 | 0.1 ± 0.2 | 0 | 0 | 0.1 ± 0.2 | 0.1 ± 0.2 | 0.2 ± 0.9 | 0 | 0.1 ± 0.2 | 0 |
| Tipulidae | 1.9 ± 1.4 | 0.3 ± 0.8 | 0 | 0.4 ± 1.1 | 0.1 ± 0.3 | 0 | 0.3 ± 0.6 | 0.1 ± 0.2 | 0 | 0.1 ± 0.3 | 0.1 ± 0.3 | 0 | 0.1 ± 0.3 |
| PHYLUM MOLLUSCA |  |  |  |  |  |  |  |  |  |  |  |  |  |
| Class Bivalvia |  |  |  |  |  |  |  |  |  |  |  |  |  |
| Corbiculidae | 0 | 0 | 0 | 0 | 0 | 0 | 0 | 0 | 0 | 0 | 0.1 ± 0.2 | 0 | 0 |
| Sphaeriidae | 0 | 0.1 ± 0.2 | 0 | 0 | 0 | 0 | 0 | 0.1 ± 0.2 | 0 | 0 | 0.3 ±0.6 | 0 | 0 |
| Class Gastropoda |  |  |  |  |  |  |  |  |  |  |  |  |  |
| Order Basommatophora |  |  |  |  |  |  |  |  |  |  |  |  |  |
| Lymnaeidae | 0 | 0 | 0 | 0 | 0.1 ± 0.5 | 0.1 ± 0.2 | 0 | 0 | 0 | 0 | 0 | 0 | 0 |
| Physidae | 0 | 0 | 5.7 ± 15.0 | 0.1 ± 0.5 | 4.1 ± 8.6 | 5.3 ± 11.5 | 2.5 ± 4.6 | 0.5 ± 2.2 | 4.0 ± 12.4 | 5.4 ± 8.8 | 63.4 ± 51.0 | 0.4 ± 1.2 | 8.4 ± 17.1 |
| Planorbiidae | 0 | 0.5 ± 1.8 | 0.4 ± 0.8 | 0.1 ± 0.2 | 1.0 ± 2.4 | 1.4 ± 3.0 | 0.5 ± 0.8 | 0.1 ± 0.2 | 0.1 ± 0.2 | 0.5 ± 1.4 | 4.7 ± 4.8 | 0.4 ± 0.8 | 0.5 ± 1.8 |
| Order Mesogastropoda |  |  |  |  |  |  |  |  |  |  |  |  |  |
| Hydrobiidae | 0.1 ± 0.3 | 0 | 0.6 ± 2.5 | 0 | 0 | 0.1 ± 0.2 | 0.1 ± 0.2 | 0 | 0 | 0.2 ± 0.5 | 0.1 ± 0.5 | 0 | 0 |
| Mean (±SE) abundance | **70.7 ± 158.9** | **87.8 ± 371.5** | **110.9 ± 478.0** | **40.5 ± 153.6** | **79.1 ± 347.0** | **75.3 ± 267.3** | **66.0 ± 282.7** | **17.0 ± 71.7** | **59.8 ± 232.5** | **59.4 ± 250.2** | **47.9 ± 194.5** | **9.0 ± 38.7** | **36.3 ± 113.6** |
| Total abundance | **4032** | **5003** | **6323** | **2308** | **4506** | **4289** | **3759** | **971** | **3409** | **3387** | **2728** | **511** | **2068** |
| Total taxon richness | **40** | **29** | **17** | **34** | **22** | **20** | **24** | **19** | **19** | **23** | **21** | **17** | **20** |
