# Supplementary material for: Effects of multiple stressors associated with agriculture on stream macroinvertebrate communities in a tropical catchment
Source: PLoS One. 2019 Aug 8;14(8):e0220528. doi: 10.1371/journal.pone.0220528 (PMC6687280; doi:10.1371/journal.pone.0220528)
Supplement: S8 Table — (DOCX) [file pone.0220528.s009.docx]

**Effects of multiple stressors associated with agriculture on stream macroinvertebrate communities in a tropical catchment**

Aydeé Cornejo, Alan M. Tonin, Brenda Checa, Ana Raquel Tuñon, Diana Pérez, Enilda Coronado, Stefani González, Tomás Ríos, Pablo Macchi, Francisco Correa-Araneda, Luz Boyero.

**Supporting information**

**S8 Table.** Average (±SD) score of biotic indices at each study site in 20 sampling campaigns.

|  | S-01 | S-02 | S-03 | S-04 | S-05 | S-06 | S-07 | S-08 | S-09 | S-10 | S-11 | S-12 | S-13 |
| --- | --- | --- | --- | --- | --- | --- | --- | --- | --- | --- | --- | --- | --- |
| SPEAR_pesticides_ | 46.2 ± 4.8 | 38.3 ± 14.0 | 35.1 ± 9.5 | 41.6 ± 13.0 | 33.5 ± 11.2 | 32.5 ± 11.6 | 21.2 ± 12.6 | 18.5 ± 18.2 | 28.4 ± 18.8 | 27.2 ± 15.0 | 6.2 ± 6.3 | 7.3 ± 16.9 | 31.0 ± 14.8 |
| BMWP/PAN | 77.5 ± 14.6 | 27.2 ± 13.0 | 28.4 ± 5.7 | 35.9 ± 11.7 | 28.6 ± 8.7 | 30.4 ± 8.1 | 26.5 ± 10.3 | 11.8 ± 8.8 | 23.4 ± 10.2 | 25.6 ± 10.4 | 23.1 ± 5.3 | 7.7 ± 7.3 | 24.6 ± 10.2 |

1999.
