# Supplementary material for: Effects of multiple stressors associated with agriculture on stream macroinvertebrate communities in a tropical catchment
Source: PLoS One. 2019 Aug 8;14(8):e0220528. doi: 10.1371/journal.pone.0220528 (PMC6687280; doi:10.1371/journal.pone.0220528)
Supplement: S9 Table — (DOCX) [file pone.0220528.s010.docx]

**Effects of multiple stressors associated with agriculture on stream macroinvertebrate communities in a tropical catchment**

Aydeé Cornejo, Alan M. Tonin, Brenda Checa, Ana Raquel Tuñon, Diana Pérez, Enilda Coronado, Stefani González, Tomás Ríos, Pablo Macchi, Francisco Correa-Araneda, Luz Boyero.

**Supporting information**

**S9 Table**. Results of the most plausible models (estimate and lower and upper 95% confidence intervals [CI]) after model selection. Plausible models (two candidate models for each response variable but abundance) were selected based on Akaike weights (*wi*, probability that a model is the “best” among the whole set of models) to account for a total probability higher than 50%. Estimates and 95% confidence intervals for each response variable were obtained using the model averaging approach. Total degrees of freedom for all models are 217. PT, pesticides (Tu_max_); SE, Sediment deposition index (inverse of sediment deposition); NE, nutrient enrichment (SRP).

| Model | Model terms | Estimate | Lower CI | Upper CI |
| --- | --- | --- | --- | --- |
| **(3)** | **Abundance** (*wi* = 0.657) |  |  |  |
|  | Intercept | 149.63 | 119.54 | 179.72 |
|  | NE | -9.56 | -22.32 | 3.20 |
|  | SE | 38.38 | 16.59 | 60.17 |
|  | PT | -25.50 | -39.39 | -11.61 |
|  | SE * PT | -13.32 | -23.82 | -2.82 |
| **(3)** | **Richness** (*wi* = 0.361) |  |  |  |
|  | Intercept | 7.64 | 6.18 | 9.11 |
|  | NE | -0.67 | -1.17 | -0.18 |
|  | SE | 0.69 | 0.05 | 1.33 |
|  | PT | -0.38 | -0.66 | -0.09 |
|  | SE * PT | -0.27 | -0.55 | 0.02 |
| **(2)** | **Richness** (*wi* = 0.236) |  |  |  |
|  | Intercept | 6.61 | 6.35 | 9.50 |
|  | NE | -5.77 | -0.96 | 0.42 |
|  | SE | 0.08 | 0.10 | 1.40 |
|  | PT | -0.19 | -0.63 | -0.07 |
|  | NE * SE | 0.39 | -0.09 | 0.87 |
| **(3 + 2)** | **Richness – Model averaging** |  |  |  |
|  | Intercept | 7.76 | 6.21 | 9.31 |
|  | NE | -0.51 | -1.22 | 0.19 |
|  | SE | 0.71 | 0.06 | 1.37 |
|  | PE | -0.36 | -0.65 | -0.08 |
|  | SE * PE | -0.27 | -0.55 | 0.02 |
|  | NE * SE | 0.39 | -0.09 | 0.87 |
| **(1)** | **SPEAR** (*wi* = 0.489) |  |  |  |
|  | Intercept | 29.22 | 26.33 | 32.12 |
|  | NE | -4.04 | -6.76 | -1.33 |
|  | SE | 7.70 | 5.17 | 10.23 |
|  | PT | -0.56 | -1.73 | 0.62 |
| **(2)** | **SPEAR** (*wi* = 0.194) |  |  |  |
|  | Intercept | 28.55 | 25.19 | 31.91 |
|  | NE | -4.81 | -8.11 | -1.52 |
|  | SE | 7.34 | 4.70 | 9.98 |
|  | PT | -0.55 | -1.72 | 0.62 |
|  | NE * SE | -0.92 | -3.25 | 1.42 |
| **(1 + 2)** | **SPEAR – Model averaging** |  |  |  |
|  | Intercept | 29.03 | 25.91 | 32.16 |
|  | NU | -4.26 | -7.26 | -1.26 |
|  | SE | 7.60 | 4.99 | 10.20 |
|  | PE | -0.55 | -1.74 | 0.63 |
|  | NE * SE | -0.92 | -3.25 | 1.42 |
| **(3)** | **BMWP** (*wi* = 0.378) |  |  |  |
|  | Intercept | 28.89 | 21.95 | 35.84 |
|  | NE | -2.94 | -4.61 | -1.27 |
|  | SE | 2.51 | 0.12 | 4.90 |
|  | PT | -1.40 | -2.43 | -0.38 |
|  | SE * PT | -0.81 | -1.74 | 0.13 |
| **(1)** | **BMWP** (*wi* = 0.316) |  |  |  |
|  | Intercept | 28.95 | 21.93 | 35.96 |
|  | NE | -3.00 | -4.66 | -1.34 |
|  | SE | 2.33 | -0.07 | 4.73 |
|  | PT | -1.14 | -2.13 | -0.15 |
| **(3 + 1)** | **BMWP – Model averaging** |  |  |  |
|  | Intercept | 28.92 | 21.86 | 35.97 |
|  | NE | -2.96 | -4.65 | -1.28 |
|  | SE | 2.43 | 0.01 | 4.85 |
|  | PT | -1.28 | -2.33 | -0.23 |
|  | SE * PT | -0.81 | -1.74 | 0.13 |
